# A Profound Basic Characterization of eIFs in Gliomas: Identifying eIF3I and 4H as Potential Novel Target Candidates in Glioma Therapy

**DOI:** 10.3390/cancers13061482

**Published:** 2021-03-23

**Authors:** Stefanie Krassnig, Christina Wohlrab, Nicole Golob-Schwarzl, Andrea Raicht, Christoph Schatz, Anna Maria Birkl-Toeglhofer, Christina Skofler, Nadine Gantenbein, Marlene Leoni, Martin Asslaber, Stefan L. Leber, Kariem Mahdy-Ali, Gord von Campe, Marlene Mayer, Andrea Borenich, Serge Weis, Martin Benesch, Johannes Haybaeck

**Affiliations:** 1 Diagnostic & Research Center for Molecular BioMedicine, Department of Neuropathology, Diagnostic and Research Institute of Pathology, Medical University Graz, Neue Stiftingtalstrasse 6, 8010 Graz, Austria; stefanie.krassnig@medunigraz.at (S.K.); christinawohlrab77@gmail.com (C.W.); nicole.golob@medunigraz.at (N.G.-S.); anna.birkl-toeglhofer@i-med.ac.at (A.M.B.-T.); christina.wodlej@medunigraz.at (C.S.); nadine.g@gmx.li (N.G.); marlene.leoni@medunigraz.at (M.L.); martin.asslaber@medunigraz.at (M.A.); stefan.leber@medunigraz.at (S.L.L.); 2 Department of Dermatology and Venereology, Medical University Graz, Auenbruggerplatz 8, 8036 Graz, Austria; 3 Department of Paediatrics and Adolescent Medicine, Division of Paediatric Haematology and Oncology, Medical University Graz, Auenbruggerplatz 38, 8036 Graz, Austria; andrea.raicht@klinikum-graz.at (A.R.); marlene.mayer@medunigraz.at (M.M.); martin.benesch@medunigraz.at (M.B.); 4 Institute of Pathology, Neuropathology and Molecular Pathology, Medical University of Innsbruck, Müllerstraße 44, 6020 Innsbruck, Austria; christoph.schatz@i-med.ac.at; 5 Center for Biomarker Research in Medicine, Stiftingtalstrasse 5, 8010 Graz, Austria; 6 Division of Neuroradiology, Vascular & Interventional Radiology, Department of Radiology, Medical University of Graz, Auenbruggerplatz 9, 8036 Graz, Austria; 7 Department of Neurosurgery, Medical University Graz, Auenbruggerplatz 29, 8036 Graz, Austria; kariem.mahdy-ali@medunigraz.at (K.M.-A.); gord.von-campe@medunigraz.at (G.v.C.); 8 Institute for Medical Informatics, Statistics and Documentation, Medical University Graz, Auenbruggerplatz 2, 8036 Graz, Austria; andrea.borenich@medunigraz.at; 9 Department of Neuropathology, Neuromed Campus Wagner-Jauregg, Kepler University Hospital, Wagner-Jauregg-Weg 15, 4020 Linz, Austria; Serge.Weis@kepleruniklinikum.at

**Keywords:** diffuse astrocytoma, anaplastic astrocytoma, glioblastoma, eukaryotic initiation factors (eIFs), mTOR signaling

## Abstract

**Simple Summary:**

Gliomas are brain tumors with currently limited therapy options. Glioma growth and proliferation is regulated by the mTOR pathway together with eukaryotic initiation factors (eIFs). In this work we show a profound basic characterization of eIFs in human gliomas and demonstrate increased mRNA and protein expressions of several eIFs in gliomas compared to healthy control brain tissue. Moreover, increased eIF3I and eIF4H levels seem to have a negative influence on the survival of patients. Our work suggests eIF3I and eIF4H as potential targets for future glioma therapy.

**Abstract:**

Glioblastoma (GBM) is an utterly devastating cerebral neoplasm and current therapies only marginally improve patients’ overall survival (OS). The PI3K/AKT/mTOR pathway participates in gliomagenesis through regulation of cell growth and proliferation. Since it is an upstream regulator of the rate-limiting translation initiation step of protein synthesis, controlled by eukaryotic initiation factors (eIFs), we aimed for a profound basic characterization of 17 eIFs to identify potential novel therapeutic targets for gliomas. Therefore, we retrospectively analyzed expressions of mTOR-related proteins and eIFs in human astrocytoma samples (WHO grades I–IV) and compared them to non-neoplastic cortical control brain tissue (CCBT) using immunoblot analyses and immunohistochemistry. We examined mRNA expression using qRT-PCR and additionally performed in silico analyses to observe the influence of eIFs on patients’ survival. Protein and mRNA expressions of eIF3B, eIF3I, eIF4A1, eIF4H, eIF5 and eIF6 were significantly increased in high grade gliomas compared to CCBT and partially in low grade gliomas. However, short OS was only associated with high eIF3I gene expression in low grade gliomas, but not in GBM. In GBM, high eIF4H gene expression significantly correlated with shorter patient survival. In conclusion, we identified eIF3I and eIF4H as the most promising targets for future therapy for glioma patients.

## 1. Introduction

Gliomas are the most common primary brain tumors originating from glial cells. According to the new 2016 WHO classification, gliomas are subdivided into astrocytic tumors, including pilocytic astrocytomas (WHO grade I), diffuse astrocytic tumors, including diffuse astrocytomas (WHO grade II), anaplastic astrocytomas (WHO grade III) and glioblastoma (GBM) (WHO grade IV) [1]. Current treatment strategies for highly malignant gliomas include surgical resection followed by adjuvant radio-chemotherapy [2]. However, with a median overall survival (OS) of 12–15 months, the outcome is still dismal [3,4]. Local, or less commonly distant, recurrence is observed in most patients following initial resection and chemo-/radiotherapy, exemplifying the tumor’s infiltrative nature and heterogeneity [5].

The PI3K/AKT/mechanistic target of rapamycin (mTOR) signaling pathway is a major survival pathway and has already been extensively studied in GBM. Deregulation of PI3K/AKT/mTOR signaling seems to be one of the key players driving gliomagenesis [6]. Common mutations detected in GBM also induce a constitutive activation of this pathway [7]. Amongst others, the PI3K/AKT/mTOR pathway regulates the rate-limiting step of protein synthesis [8].

Translation from mRNA into protein can be divided into four steps, denoted as initiation, elongation, termination and ribosome recycling. Translation is mainly regulated at the initiation step and is governed by eukaryotic initiation factors (eIFs). The initiation process starts with the formation of the 43S preinitiation complex (PIC) under the assistance of the eIF3 complex, eIF1 and eIF1A [9,10]. In the PI3K/AKT/mTOR-dependent eIF4F complex activation, phosphorylation of eukaryotic translation initiation factor 4E-binding protein 1 (4E-BP1) leads to the formation of the eIF4F complex consisting of eIF4A, eIF4E and eIF4G [11]. The eIF4F complex recognizes the 7-methyl-GTP cap structure at the 5′ mRNA end and joins the PIC. Afterwards, the scanning for the AUG start codon and formation of the mature 80S ribosome takes place [9,10].

eIFs may serve as tumor suppressors or promote carcinogenesis as well as tumor progression in different types of cancer [12]. Alterations in the eIF signaling cascade have also been reported in previous glioma studies. In vitro silencing or inhibition of eIF3B [13], eIF3C [14], eIF3D [15] and eIF3E [16] reduced cell proliferation and increased apoptosis in GBM cell lines. Overexpression of eIF3C [14], eIF3D [15] and eIF5A [17] was described in human glioma tissues, predominantly in WHO grades III and IV. Phospho (p)-eIF4E and p-4E-BP1 levels were increased during malignant progression in astrocytoma [18,19]. Treatment of U87MG cells and U87MG-derived xenografts with the eIF4F-complex formation inhibitor 4EGI-1 decreased cell growth and induced apoptosis and mitochondrial dysfunction [20]. Recently, in silico analyses from The Cancer Genome Atlas (TCGA) and the Chinese Glioma Genome Atlas (CGGA) revealed a significant impact of various eIF3 subunits in astrocytomas (WHO grades I–IV). In particular, the prognostic value of eIF3I gene expression in IDH1/2 mutant lower grade gliomas was shown [21].

The majority of eIF subunits have not been investigated so far, or only at the protein or mRNA level. The aim of the present study was to bridge this gap and to characterize a broad panel of eIFs in terms of protein and mRNA levels in astrocytomas (WHO grades I–IV), followed by their correlation with OS in two different publicly available databases.

## 2. Results

### 2.1. Active PI3K/AKT/mTOR Signaling Pathway and Regulation of Translational Initiation in Gliomas

**Figure 1:**
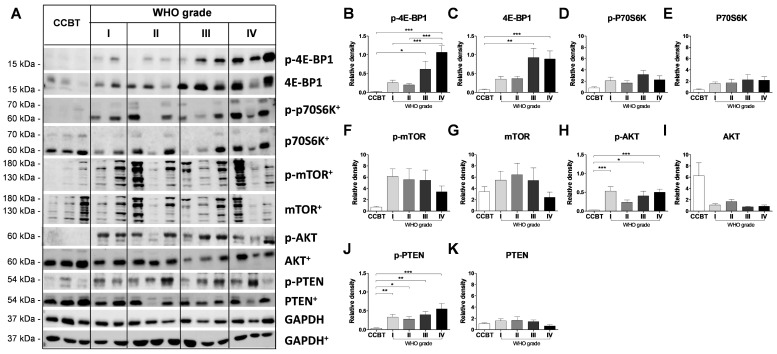
Active PI3K/AKT/mTOR signaling pathway and regulation of translational initiation in astrocytomas (WHO grades I–IV). Representative immunoblots for PI3K/AKT/mTOR signaling expression are shown (**A**). Densitometric analysis of immunoblots was performed using ImageJ software (NIH, MD, United States). For relative densities, expression of p-4E-BP1 (**B**), 4E-BP1 (**C**), p-p70S6K (**D**), p70S6K (**E**), p-mTOR (**F**), mTOR (**G**), p-AKT (**H**), AKT (**I**), p-PTEN (**J**) and PTEN (**K**) was normalized to the loading control (GAPDH). Immunoblot images marked with a cross were performed on an 8% SDS gel and immunoblot images without any marks on a 12.5% SDS gel. As the size of tumor tissue in particular for GBM samples was limited, several markers from the eIF and PI3K/mTOR/AKT signaling pathway were analyzed on the same immunoblot. Membranes were stripped several times with a stripping buffer and afterwards incubated with another antibody. Therefore, GAPDH+ images for 8% SDS gels are identical in Figure 1 and Figure 2 as eIFs (eI6, eIF1A, eIF3I, eIF4A, p-eIF4G, eIF4H, IDH1) as well as members of the PI3K/AKT/mTOR pathway (p-4EBP-1, 4EBP-1, p-AKT and p-PTEN) were analyzed on the same immunoblot. Numbers: CCBT (white bars): *n* = 6–12; pilocytic astrocytoma (WHO grade I; light grey bars): *n* = 6–10, diffuse astrocytoma (WHO grade II; grey bars): *n* = 8–13, anaplastic astrocytoma (WHO grade III; dark grey bars): *n* = 6–8, GBM (WHO grade IV, black bars): *n* = 8–13. Bars represent group means + SEM. Statistical analysis: one-way ANOVA with Bonferroni or Dunn’s post-test. Significance levels: *** *p* < 0.001; ** *p* < 0.01, * *p* < 0.05. Abbreviations: 4E-BP1, eIF4E-binding protein 1; AKT, protein kinase B; ANOVA, analysis of variance; CCBT, non-neoplastic cortical control brain tissue; GAPDH, glyceraldehyde 3-phosphate dehydrogenase; mTOR, mammalian/mechanistic target of rapamycin; p70S6K, p70 ribosomal protein S6 kinase; PTEN, phosphatase and tensin homologue; SEM, standard error of means.

First, we aimed to confirm the activation of the PI3K/AKT/mTOR pathway using immunoblot analyses (Figure 1). The expression of 4E-BP1 (Figure 1B) and its phosphorylated form (Figure 1C) increased stepwise from low grade gliomas (LGGs) to GBM compared to non-neoplastic cortical control brain tissue (CCBT). Expression of p-4E-BP1 was significantly higher compared to CCBT (III: *p* < 0.05, IV: *p* < 0.001). In addition, the p-4E-BP1 protein expression differed significantly between LGGs and GBM (II: *p* < 0.001, III: *p* < 0.001; Figure 1B). Significantly increased protein expression was also detected for p-AKT (I: *p* < 0.001, III: *p* < 0.05, IV: *p* < 0.001; Figure 1H) and p-PTEN (I: *p* < 0.01, II: *p* < 0.05, III: *p* < 0.01, IV: *p* < 0.001; Figure 1J). p-p70S6K (Figure 1D) and p-mTOR (Figure 1F), were not increased significantly compared to controls.

### 2.2. Basic Characterization of eIF Protein Expression in Human Astrocytomas (WHO Grade I–IV)

**Figure 2:**
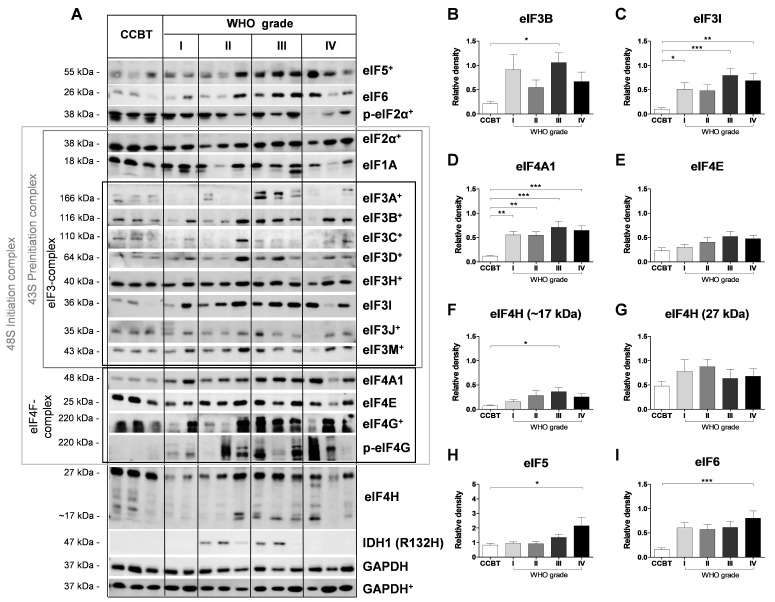
Basic characterization of various eukaryotic initiation factors (eIFs) for protein level in astrocytomas (WHO grades I–IV) compared to CCBT. eIF protein expression was analyzed using immunoblot analysis. Representative immunoblots for eIF5, eIF6, (p-) eIF2α, eIF1A, eIF3 subunits (A, B, C, D, H, I, J, M), eIF4F complex members (4A1, 4E, (p-)4G), eIF4H and IDH1 (R132H) are shown (**A**). Densitometric analysis of immunoblots was performed using ImageJ software (NIH, MD, United States). For relative densities, expression of eIF3B (**B**), eIF3I (**C**), eIF4A (**D**), eIF4E (**E**), eIF4H (approximately 17 kDa) (**F**), eIF4H (27 kDa) (**G**), eIF5 (**H**) and eIF6 (**I**) was normalized to the loading control (GAPDH). Immunoblot images marked with a cross were performed on an 8% SDS gel, immunoblot images without any marks on a 12.5% SDS gel. As the size of tumor tissue in particular for GBM samples was limited, several markers from the eIF and PI3K/mTOR/AKT signaling pathway were analyzed on the same immunoblot. Membranes were stripped several times with a stripping buffer and afterwards incubated with another antibody. Therefore, GAPDH+ images for 8% SDS gels are identical in Figure 1 and Figure 2 as eIFs (eI6, eIF1A, eIF3I, eIF4A, p-eIF4G, eIF4H, IDH1) as well as members of the PI3K/AKT/mTOR pathway (p-4EBP-1, 4EBP-1, p-AKT and p-PTEN) were analyzed on the same immunoblot. Bars represent group means + SEM. Numbers: CCBT (white bars): *n* = 6–12; pilocytic astrocytoma (WHO grade I; light grey bars): *n* = 6–10, diffuse astrocytoma (WHO grade II; grey bars): *n* = 6–13, anaplastic astrocytoma (WHO grade III; dark grey bars): *n* = 6–8, GBM (WHO grade IV, black bars): *n* = 6–13. Statistical analysis: one-way ANOVA with Bonferroni post-test. Significance levels: *** *p* < 0.001; ** *p* < 0.01, * *p* < 0.05. *Abbreviations: ANOVA, analysis of variance; CCBT, non-neoplastic cortical control brain tissue; GAPDH, glyceraldehyde 3-phosphate dehydrogenase; IDH1 (R132H), isocitrate dehydrogenase 1 (arginine to histidine substitution at position 132);* kDa, kilodalton; *SEM, standard error of means.*

eIF1A and eIF2α showed no difference in their protein expression in gliomas compared to CCBT (Figure 2A and Figure S1A,C). p-eIF2α was significantly increased only in pilocytic astrocytoma (*p* < 0.05; Figure S1B).

Regarding the eIF3 complex, significantly higher protein expression in gliomas compared to CCBT was detected for eIF3B (III: *p* < 0.05; Figure 2B), eIF3D (I: *p* < 0.05; III: *p* < 0.05; Figure S1F), eIF3H (III: *p* < 0.05; Figure S1G), eIF3I (I: *p* < 0.05, III: *p* < 0.001, IV: *p* < 0.01; Figure 2C) and eIF3M (III: *p* < 0.01; Figure S1I). However, only eIF3I showed a stepwise increase in protein expression over all four tumor grades (Figure 2C); protein expression of the other eIF3-subunits varied between the tumor grades. No differences were observed for eIF3A (Figure S1D), eIF3C (Figure S1E) and eIF3J (Figure S1H).

eIF4F complex members also showed altered protein expression in astrocytomas. Significantly elevated protein levels were found for eIF4A1 (I: *p* < 0.01, II: *p* < 0.01, III: *p* < 0.001, IV: *p* < 0.001; Figure 2D) and p-eIF4G (I: *p* < 0.01, II: *p* < 0.05, III: *p* < 0.01, IV: *p* < 0.01; Figure S1J) in astrocytomas compared to CCBT. However, the expression of p-eIF4G and eIF4G varied within the astrocytoma samples. eIF4E protein levels increased over the four tumor grades (Figure 2E). eIF4H showed two bands at 27 kDa (Figure 2F) and approximately 17 kDa (Figure 2G).Whereas the 27 kDa band did not show any differences compared to CCBT, the band at approximately 17 kDa was only present in astrocytoma samples (III: *p* < 0.05). eIF5 (*p* < 0.05; Figure 2H) and eIF6 (*p* < 0.001; Figure 2I) protein levels also varied within astrocytoma sample and significantly increased protein levels were only detected in GBM patients compared to CCBT.

### 2.3. mRNA Expression of Selected eIFs in Astrocytomas (WHO Grades I–IV)

**Figure 3:**
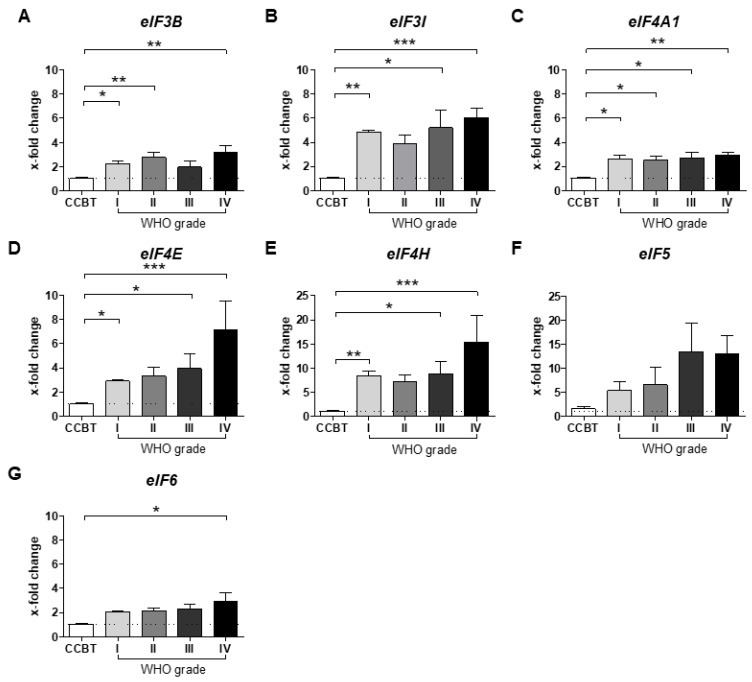
mRNA expression of selected eIFs in astrocytomas (WHO grades I–IV) compared to CCBT. mRNA expression of *eIF3B* (**A**), *eIF3I* (**B**), *eIF4A1* (**C**), *eIF4E* (**D**), *eIF4H* (**E**), *eIF5* (**F**) and *eIF6* (**G**) was analyzed in astrocytomas (WHO grades I–IV) compared to CCBT using qRT-PCR. mRNA levels are shown as x-fold change to CCBT calculated with the ΔΔC(t) method. *GAPDH* and *SDHA* were used as housekeeping genes. CCBT (white bars): *n* = 5–7; pilocytic astrocytoma (WHO grade I; light grey bars): *n* = 7, diffuse astrocytoma (WHO grade II; grey bars): *n* = 9, anaplastic astrocytoma (WHO grade III; dark grey bars): *n* = 6, GBM (WHO grade IV, black bars): *n* = 11–15. Statistical analysis: one-way ANOVA with Bonferroni post-test. Significance levels: *** *p* < 0.001; ** *p* < 0.01, * *p* < 0.05. Abbreviations: ANOVA, analysis of variance; CCBT, non-neoplastic cortical control brain tissue; GAPDH, glyceraldehyde 3-phosphate dehydrogenase; SDHA, succinate dehydrogenase complex flavoprotein subunit A; SEM, standard error of means.

Next, mRNA expression was determined for *eIF3B* (I: *p* < 0.05, II: *p* < 0.01, IV: *p* < 0.01; Figure 3A), *eIF3I* (I: *p* < 0.01, III: *p* < 0.05, IV: *p* < 0.001; Figure 3B), *eIF4A1* (I: *p* < 0.05, III: *p* < 0.05, III: *p* < 0.05, IV: *p* < 0.01; Figure 3C), *eIF4E* (I: *p* < 0.05, III: *p* < 0.05, IV: *p* < 0.001; Figure 3D) and *eIF4H* (I: *p* < 0.01, III: *p* < 0.05, IV: *p* < 0.001; Figure 3E). mRNA levels were significantly higher in all tumor grades compared to CCBT. *eIF5* (Figure 3F) expression showed a high variability throughout all samples and *eIF6* (IV: *p* < 0.01; Figure 3G) expression was only elevated in high grade gliomas (HGGs) compared to CCBT.

### 2.4. Impact of eIF Gene Expression on Patients’ Overall Survival

**Figure 4:**
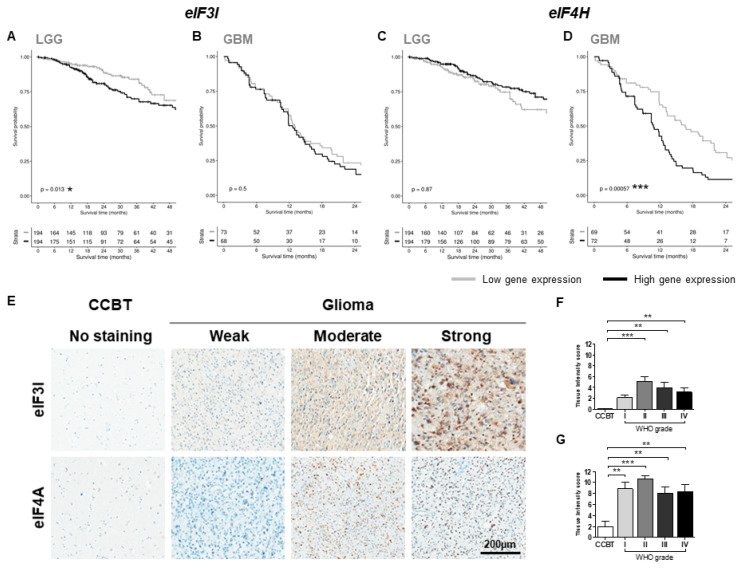
Survival analysis and immunohistochemical evaluation of eIF3I and eIF4H. Gene expression data for survival analysis from the TCGA database were divided into low or high gene expression groups according to the gene expression level of the median. Survival of those two groups was compared using the log-rank test. Graphs represent Kaplan–Meier curves of *eIF3I* (**A**,**B**) and *eIF4H* (**C**,**D**) in LGG (WHO grade II; (**A**,**C**)) and GBM (WHO grade IV; (**B**,**D**)). Numbers: WHO grades I–II = 388, WHO grade IV = 141. Significance levels: * *p* < 0.05; *** *p* < 0.001. Representative pictures for weak, moderate and strong staining of eIF3I and eIF4H in CCBT and GBM (WHO grade IV) (**E**). Graphs show the tissue intensity score of eIF3I (**F**) and eIF4H (**G**). Bars represent group means + SEM. Numbers: CCBT (white bars): *n* = 14, 15, pilocytic astrocytoma (WHO grade I; light grey bars): *n* = 18, 19, diffuse astrocytoma (WHO grade II; grey bars): *n* = 22, 24, anaplastic astrocytoma (WHO grade III; dark grey bars): *n* = 18, 23, GBM (WHO grade IV; black bars): *n* = 20, 16. Statistical analyses: one-way ANOVA followed by Dunn’s post-hoc test. Significance levels: * *p* < 0.05; ** *p* < 0.01, *** *p* < 0.001. Abbreviations: ANOVA, analysis of variance; CCBT, non-neoplastic cortical control brain tissue; LGG, low grade glioma; SEM, standard error of means.

To evaluate the impact of eIF gene expression on the OS of patients with glioma, the publicly available gene expression datasets from the TCGA database were analyzed. Two TCGA datasets were studied: LGGs (WHO grades I–II) and GBM (WHO grade IV). Kaplan–Meier curves were generated for *eIF3B*, *eIF3I*, *eIF4A1*, *eIF4H*, *eIF4E, eIF5* and *eIF6* (Figure 4 and Figure S2).

Only eIF3I and eIF4H were significantly associated with patient outcome. Higher *eIF3I* expression was significantly associated with reduced OS in LGG patients (*p* < 0.05; Figure 4A) but did not affect the survival of GBM patients (Figure 4B). *eIF4H* (*p* < 0.001; Figure 4D) was negatively correlated with the OS of GBM patients, but not of patients with LGGs (Figure 4C). *eIF3B* (Figure S2A) and *eIF4E* (Figure S2E) showed a tendency to be associated with a reduced OS for LGG patients and *eIF3B* (Figure S2F) and *eIF4A1* (Figure S2G) for GBM patients. Gene expression of *eIF5* (Figure S2D,I) and *eIF6* (Figure S2E,J) was not associated with decreased OS.

An additional analysis with two independent datasets (TCGA and CGGA) comparing survival in tumors with high or low gene expression revealed a significant survival difference for *eIF3I* in TCGA data but not CGGA data (Table S6). Survival differences for *eIF4H* were significant in both TCGA and CGGA data (Table S6). Notably, *eIF6* showed significant differences in patient survival in CGGA but not TCGA data (Table S6). Additional correlation coefficients for eIF3I, eIF4H and other relevant genes in gliomas are provided in the supplementary material (Tables S4 and S5).

### 2.5. Immunohistochemical Evaluation Confirmed Increased eIF3I and eIF4H Levels

Cell type-specific protein expression of eIF3I and eIF4H was evaluated using IHC (Figure 4E). Scoring of positive cells was exclusively performed for tumor tissue; necrotic areas and infiltration zones were not scored. eIF3I was significantly increased in WHO grades II–IV tumors compared to CCBT (II: *p* < 0.001, III: *p* < 0.01, IV: *p* < 0.01; Figure 4F). eIF4H-positive cells were only detected in astrocytoma samples and were almost absent in CCBT (I: *p* < 0.01, II: *p* < 0.001, III: *p* < 0.01, IV: *p* < 0.01; Figure 4G). The IDH1 (R132H) mutation had no significant impact on expression status of eIF3I or eIF4H positive tumor cells (Figure S3).Error bars are partly missing because of the low number of samples or similar tissue intensity scores in the immunohistochemical evaluation.

Finally, immunohistochemical results for eIF3I and eIF4H were correlated with patients’ relapse-free survival and OS in LGGs (WHO grades I–II) and HGGs (WHO grades II–IV, Figure S4). As group numbers were too low to determine hazard ratios, only proportion scores were generated (Table 1).

## 3. Discussion

Gliomas are very heterogeneous tumors with varying clinical outcomes. Although much effort has gone into the development of novel therapies over the last few years, treatment options are still very limited and many clinical trials have failed [22]. New biomarkers and therapeutic approaches are urgently required. Thus, the purpose of our study was to identify eIFs that can serve as novel prognostic biomarkers or therapeutic targets in gliomas.

Amongst others, the regulation of cell proliferation and cell survival is one major task of the PI3K/AKT/mTOR signaling pathway. Dysregulation of these functions belongs to the disease characteristics of cancer and the PI3K/AKT/mTOR pathway has been identified as a key player driving carcinogenesis in various tumor entities such as breast cancer and renal cell carcinoma [23]. Within this study, the protein expression of PI3K/AKT/mTOR pathway members was analyzed and activation of this pathway in astrocytomas could be confirmed [24,25,26]. We found a significantly increased protein expression of p-AKT and p-PTEN in gliomas compared to CCBT. Increased expression levels of mTOR pathway members have been already extensively reported previously [24,25,26].

**Figure 5:**
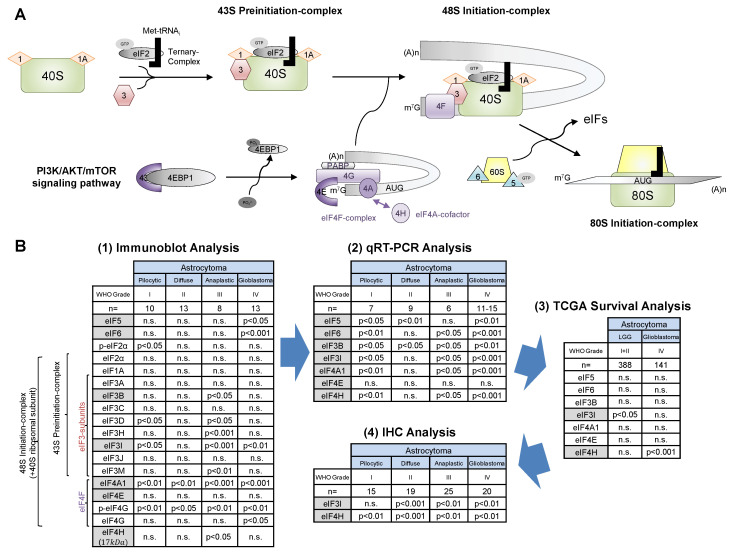
Summary of the eIF basic characterization in astrocytomas (WHO grades I–IV). Illustration of eukaryotic translation initiation (**A**). Translation initiation begins with the formation of the 43S preinitiation complex in which the 40S ribosomal subunit associates with a ternary complex (eIF2-GTP-Met-tRNAi) with the support of eIF1, eIF1A and the eIF3 complex (including eIF3I). For cap-dependent translation initiation, activation of the PI3K/AKT/mTOR signaling pathway initiates the dissociation of 4E-BP1 and eIF4E via the phosphorylation of 4E-BP1, allowing eIF4F complex formation. eIF4H is a co-factor of eIF4A and stimulates its helicase activity. The eIF4F complex (eIF4A, eIF4E and eIF4G) recognizes the m^7^G cap of the mRNA and associates via eIF4G with the eIF3 of the PIC. This leads to the formation of the 48S initiation complex. After the scanning process for the AUG start codon, the 60S ribosomal subunit joins the 48S initiation complex under the assistance of eIF5 and eIF6. eIF5-mediated hydrolysis of the ternary complex leads to the dissociation of the eIFs and the formation of an elongation-competent 80S ribosome. Schematic diagram of the search for novel eIF biomarkers in astrocytomas of WHO grades I–IV (**B**). For each analysis, numbers of individuals included and analyzed eIFs are listed. *p*-values listed in the table reveal significant differences between the respective tumor grade and CCBT. (1) First, 18 eIF subunits were investigated using immunoblot analyses. (2) Next, 7 out of 18 eIFs (marked in grey) were selected for further qRT-PCR analyses based on significant differences between CCBT and astrocytomas (WHO grades I–IV). (3) All 7 eIFs (marked in grey) were additionally analyzed regarding their impact on patients’ OS. (3) eIF3I and eIF4H significantly influenced the OS glioma patients and therefore were finally analyzed immunohistochemically. *Abbreviations: CCBT, non-neoplastic cortical control brain tissue; LGG, low grade glioma; PIC, preinitiation complex.*

As consequence of the deregulation of the PI3K/AKT/mTOR signaling in gliomas, the eIF signaling cascade might also play an important role during gliomagenesis (Figure 5A). 4E-BP1, the link between those two pathways, has been shown to correlate with the astrocytoma grade, as 4E-BP1 was significantly higher expressed in HGG compared to LGG [18], which was confirmed by our data.

Several eIFs have already been identified as biomarkers for various cancer entities including gliomas [12]. However, research has been mainly focused on the investigation of eIF3 subunits. We wanted to close the gap and analyze not only eIF3 subunits, but also the eIF4F complex, and other eIF subunits. Additionally, we wanted to investigate protein as well as mRNA levels to get a detailed overview on eIF expression patterns in gliomas. For the basic characterization, we compared eIF expression levels of astrocytomas to CCBT. CCBT reveals a non-uniform distribution of cells and neurons and does not only contain astrocytoma cells of origin. CCBT has already served as control in previous studies [14,19].

In the course of the basic characterization, previous alterations in the eIF signaling cascade were confirmed and additional eIFs altered in gliomas were identified. Upregulation of eIF3B [13] has been described previously in glioma patient samples. eIF3C [14], eIF3D [15] and eIF4E [18] even correlated with tumor grade as their expression was significantly higher in HGG than in LGG. In silico, increased gene expression over all four tumor grades was determined for *eIF3B*, *eIF3I*, *eIF3K* and *eIF3M* [21]. Additionally, those four factors have been associated with poorer OS in glioma patients [21]. We were able to confirm a significantly increased protein expression of eIF3B and eIF3D. For eIF3C and eIF4E an increase in the protein and mRNA levels was found; however, this was not statistically significant. Besides the already known eIFs, we demonstrated significantly elevated protein levels of eIF3D, eIF3H, eIF3I, eIF3M, p-eIF4G, eIF4H, eIF5 and eIF6 in astrocytoma tissue compared to CCBT. Notably some of these findings were not consistent in Western blot and immunohistochemistry. Differing results in protein expression might partly be explained by methodological differences. However, these findings have to be interpreted carefully and their biological significance needs to be in the focus of future research.

Based on significant differences between astrocytomas (WHO grades I–IV) and CCBT, we started a selection process for promising eIFs that could serve as novel therapeutic targets or prognostic biomarkers (Figure 5B). After immunoblot analysis, eIF3B, eIF3I, eIF4A1, eIF4H, eIF5 and eIF6 were selected for further detailed analyses. All six subunits were also significantly upregulated in mRNA level. However, eIF3I and eIF4H were the only analyzed eIFs that were significantly associated with survival differences of glioma patients. Therefore, eIF3I and eIF4H were chosen as most promising candidates for more additional immunohistochemical analysis.

eIF3I is a member of the eIF3 complex and was at first identified as TGF-β receptor II interacting protein 1 (Trip1) [27,28] and was shown to promote cell proliferation and angiogenesis in zebrafish embryos [29]. eIF3I was previously identified as a prognostic biomarker in IDH1/2 mutant LGG based on TCGA and CGGA database analyses [21]. We were able to confirm its impact on the OS of LGG patients in the TCGA database. Additionally, we also showed for the first time a stepwise increase of eIF3I expression over all four tumor grades in protein level.

Expression of eIF4H was significantly increased for mRNA level and protein level using immunohistochemistry. Interestingly, immunoblot analyses revealed an additional band at approximately 17 kDa, only present in astrocytoma samples. The product at 17 kDa might have been a degraded form of eIF4H or an astrocytoma-specific splicing variant. Tumor type-specific eIF4H splicing variant expression was described previously for colon cancer cell lines [30]. However, this has to be clarified in further analyses. Additionally, high eIF4H gene expression was negatively associated with the OS of GBM patients. eIF4H is an RNA binding protein that interacts with eIF4A, thereby stimulating its helicase activity [31,32]. As eIF4H and its interaction partner eIF4A1 seem to be relevant in gliomas, inhibiting the interaction between those two factors might be a novel approach in glioma therapy.

## 4. Materials and Methods

### 4.1. Patient Samples

The study was reviewed and approved by the institutional ethics committee of the Medical University of Graz (MUG) according to Austrian and European law (24–402 ex 11/12). As controls for biochemical and immunohistochemical (IHC) analysis, non-neoplastic (“healthy”) cortical control brain tissue was collected post-mortem at the Department of Pathology of the MUG (biochemical analyses: *n* = 12, immunohistochemistry: *n* = 15). Astrocytoma samples for biochemical (pilocytic astrocytoma WHO grade I: *n* = 10, diffuse astrocytoma WHO grade II: *n* = 13, anaplastic astrocytoma WHO grade III: *n* = 8, GBM WHO grade IV: *n* = 13) and immunohistochemical (pilocytic astrocytoma WHO grade I: *n* = 18, diffuse astrocytoma WHO grade II: *n* = 26, anaplastic astrocytoma WHO grade III: *n* = 22, GBM WHO grade IV: *n* = 22) analysis were obtained retrospectively (before 2016) from the Biobank of the MUG and the Brain Biobank of the Division of Neuropathology, Neuromed Campus, Kepler University Linz, Austria. As all samples were collected retrospectively before 2016, they were neuropathologically classified by board-certified neuropathologists (J.H. and S.W.) according to the 2007 WHO classification [33]. Information about age, gender and IDH mutation status was collected for immunohistochemical analysis (Table S1).

### 4.2. Tissue Processing for Biochemical Analyses

Human tissue samples were collected during frozen section, immediately frozen in liquid nitrogen and stored until further biochemical processing. Frozen tissue samples were homogenized in NP-40 lysis buffer (0.05 M Tris-HCl, 5 mM NaCl, 0.5% NP-40, 0.1 mM Pefabloc^®^, 1 mM DTT) supplemented with cOmplete™ Protease Inhibitor Cocktail and PhosSTOP™ phosphatase inhibitor using the MagNA Lyser homogenizer (all Roche Diagnostics, Risch-Rotkreuz, Switzerland). Protein concentration was determined using the Bradford protein assay (Bio-Rad Laboratories GmbH, Munich, Germany). NP-40 lysates were used for protein and mRNA analyses.

### 4.3. RNA Isolation & qRT-PCR

RNA was isolated from total protein lysates with TRIzol^®^ reagent (Life Technologies, Karlsbad, CA, USA) as described previously [34]. Oligonucleotides (Table S2) were self-designed using Primer-BLAST [35] and synthesized by Eurofins Genomics (Ebersberg, Germany). Efficiencies of all self-designed primers were calculated with known cDNA concentrations. Glyceraldehyde-3-phosphate dehydrogenase (GAPDH) and succinate dehydrogenase complex subunit A (SDHA) were used as housekeeping genes. Relative gene expression was calculated using the 2^∆∆CT^ analysis method [36].

### 4.4. Immunoblot Analysis

Immunoblot analysis was performed as described previously [34]. Briefly, total protein lysates (30 µg/sample) were used for SDS gel electrophoresis. Primary antibodies were incubated in 5% bovine serum albumin (BSA) in TBS-T (antibody dilutions listed in Table S3) over night at 4°C. Incubation with a horseradish peroxidase conjugated secondary antibody (dilutions: anti-mouse 1:3000, anti-rabbit 1:5000; GE Healthcare Life Sciences, Buckinghamshire, UK) was carried out for 1 h at room temperature. For membrane stripping, immunoblots were incubated in Restore PLUS Western blot stripping buffer (Thermo Fisher Scientific, Waltham, MA, USA) for 50 min at room temperature under shaking conditions. Semi-quantitative evaluation of immunoblots was performed using ImageJ software for densitometric analyses [37]. Relative densities were calculated by normalizing density values for each protein to the GAPDH loading control.

### 4.5. Immunohistochemistry

Immunohistochemistry for eIF3I and IDH1 (R132H) was performed on a Ventana Immunostainer XT using an UltraView DAB Detection-Kit (Ventana Medical Systems, Tucson, AZ, USA), CC1 mild as epitope retrieval and different dilutions of the antibodies (+). IHC staining was neuropathologically assessed by a board-certified neuropathologist and a second independent assessor (J.H. and M.L.) blinded to the clinical data using light microscopy. For the evaluation of the eIF expression, staining intensities (intensity score 0–3; 0, no staining, 1, weak, 2, moderate and 3, strong) and the percentage of positive cells (density score; 0–100%) were determined. Afterwards, a numeric scale was adapted to the density scores (proportion score): 0% = “0”; ≤10% = “1”; <49% = “2”; <79% = “3”; ≥80% = “4”. The tissue intensity score (TIS) was then calculated as described previously [38].

### 4.6. Patient Overall Survival Analysis

eIF gene expression and patient data from the The Cancer Genome Atlas database were downloaded from the Genomic Data Commons (GDC) (https://gdc.cancer.gov/ (accessed on 5 June 2020)). Upper quartile normalized gene expression data from “Brain Lower Grade Glioma (LGG)” (516 cases; last updated 2 May 2016) and “GBM” (528 cases; last updated 27 May 2016) were used for the analyses. Samples were categorized into high and low gene expression with expression values above or below the median, respectively. Kaplan–Meier survival curves were generated for every gene using the survival package [39] and survminer package [40] in R 3.3.0 [41]. The survival curves of patients with high and low gene expression were compared by the log-rank test. The significance level was set at *p* < 0.05.

Additional datasets from TCGA (GBMLGG) and from CGGA (693) were used for validation. Oligodrendroglioma data was removed from the TCGA datasets. Groups based on gene expression were dichotomized. A mutation for a specific gene was considered as a high expression for TCGA data. For CGGA data the expression for a gene was weighted by the clinical information if an IDH, respectively a 1p19q co-deletion, occurred. The OS of the group with the higher expression of EIF3I, respectively EIF4H, was compared to the group with the higher expression of ATRX, CDKN2A, EGFR, EIF4A1, EIF4H, EIF5, EIF6, IDH1, IDH2, MAP2, MGMT, NF1, PDGFRA, PIK3R1, PTEN and TP53. EIF3I and EIF4H were combined and the OS of the highest expressions were used, independent of the data taken from EIF3I or EIF4H. The OS from the group with the upper half expression was compared to the OS of the group with the lower half and *p*-values from the group comparisons were calculated. C# scripts were used to process the data. Using survcorr from the R package SurvCorr, the rho correlation coefficient between the survival curves was calculated.

### 4.7. Statistical Analysis

Statistical analyses and graphs were generated using Graph Pad Prism 4.03 (GraphPad Software Inc., San Diego, CA, USA). All data were tested for normal distribution using the Kolmogorov–Smirnoff test. For evaluation of biochemical and imm unohistochemical data, one-way analysis of variance (ANOVA) was used followed by Bonferroni’s multiple comparison test (normally distributed data), or the Kruskal–Wallis test followed by Dunn’s multiple comparison test (not normally distributed data), to analyze the eIF expression in astrocytomas of WHO grades I–V compared to control tissue. Kaplan–Meier curves of patients with high and low gene expression were compared with the log-rank test. Significance levels were set at *p* < 0.05. Proportion scores were calculated to analyze the impact of eIF protein expression on patients’ OS.

## 5. Conclusions

In conclusion, our study provides a profound basic characterization of various eIFs in astrocytomas of WHO grades I–IV. We showed that the expression of eIF3B, eIF3I, eIF4A1, eIF4H and eIF6 was significantly increased in astrocytomas, in particular in GBM, for protein and mRNA levels. However, the OS of glioma patients only correlated for eIF3I with LGG and for eIF4H with GBM. Therefore, targeting of eIF3I and eIF4H might represent a prospective approach towards improvement of glioma therapy. However, to finally establish eIF3I and eIF4H as therapeutic targets, gain- and loss-of-function experiments are required to assess detailed pathophysiological consequences.

## Figures and Tables

**Table 1:** Proportions for patients’ relapse-free and overall survival (3 and 5 years) for low grade gliomas (LGGs) (WHO grades I–II) and high grade gliomas (HGGs) (III–IV). Abbreviations: f, female; IDH, isocitrate dehydrogenase 1 (arginine to histidine substitution at position 132); m, male; TIS, tissue intensity score; wt, wildtype.

Variable	Category	3 Years Grade I/II	3 Years Grade III/IV	5 Years Grade I/II	5 Years Grade III/IV
**Age**	<=47	3/32	3/11	3/32	4/11
	>=48	3/13	22/31	4/13	25/31
**Gender**	m	5/26	13/22	5/26	15/22
	w	1/19	12/20	2/19	14/20
**IDH1_Status**	Mutated	1/17	3/11	1/17	4/11
	wt	3/21	20/21	3/21	21/21
**eIF3I_TIS**	0, 1, 2	4/19	12/18	4/19	14/18
	3, 4, 6	0/9	7/8	0/9	7/8
	8, 9, 12	0/7	4/6	0/7	4/6
**eIF4H_TIS**	0, 1, 2	1/2	2/5	1/2	2/5
	3, 4, 6	1/6	2/5	1/6	3/5
	8, 9, 12	1/23	9/18	2/23	11/18

## Data Availability

Original data are stored at the ‘Mendeley Data’ and available in [http://dx.doi.org/10.17632/b78ngg5xrh.1] (last edit 25 March 2021).

## Supplementary Materials

The following are available online at https://www.mdpi.com/2072-6694/13/6/1482/s1: Figure S1: Densitometric analysis of eIF immunoblots in astrocytomas (WHO grades I–IV) compared to CCBT. Figure S2: Influence of *eIF* gene expression on patients’ OS in LGG (WHO grades I–II) and GBM (WHO grade IV). Figure S3: Immunohistochemical evaluation of eIF3I and eIF4H protein expression in astrocytomas (WHO grades I–IV) compared to CCBT in consideration of the *IDH1* (R132H) mutation status. Figure S4: Influence of eIF protein expression on patients’ OS in LGG (WHO grades I–II) and HGG (WHO grades III–IV). Table S1: Descriptive statistics of clinicopathological characteristics and immunohistochemical evaluation. Table S2: Oligonucleotides used for qRT-PCR analyses. Table S3: Primary antibodies used for immunoblot and immunohistochemical analyses. Antibody dilutions for immunohistochemical analyses are listed in parentheses. Table S4: Correlation coefficients comparing EIF3I and EIF4H with glioma-relevant genes. Table S5: Correlation coefficients of high and low expression of glioma-relevant genes. Table S6: Survival comparison with *p*-values of high vs. low expression of glioma-relevant genes.
