# Supplementary material for: A Profound Basic Characterization of eIFs in Gliomas: Identifying eIF3I and 4H as Potential Novel Target Candidates in Glioma Therapy
Source: Cancers (Basel). 2021 Mar 23;13(6):1482. doi: 10.3390/cancers13061482 (PMC8004965; doi:10.3390/cancers13061482)
Supplement: Supplementary file 1 [file cancers-13-01482-s001.zip › Final Revision FiguresGlioma_manuscript ID (cancers-844614).pptx]

## Slide 1
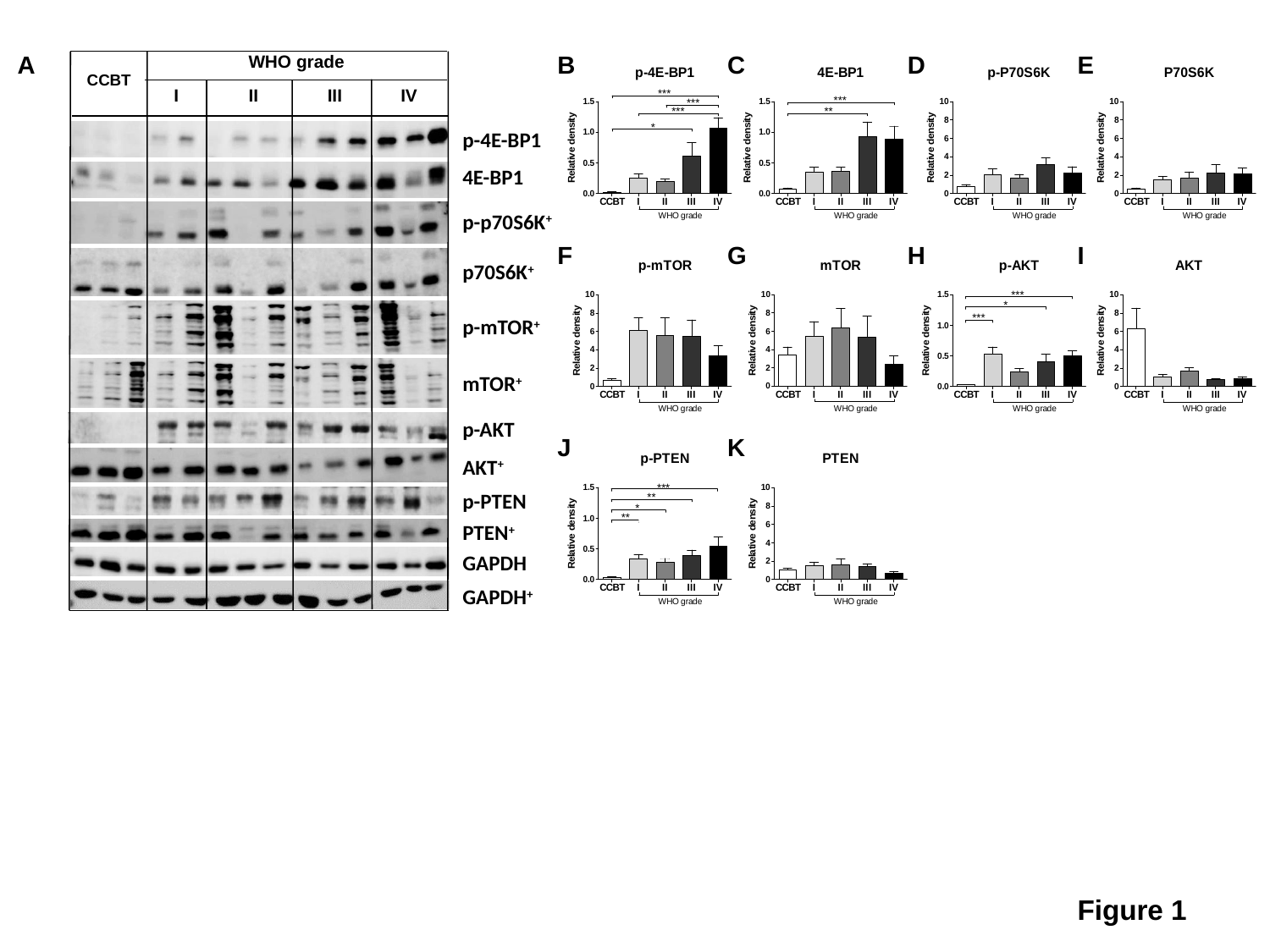

A
B
C
D
E
| CCBT | WHO grade | | | |
| --- | --- | --- | --- | --- |
| | I | II | III | IV |
p-4E-BP1
4E-BP1
p-p70S6K+
F
G
H
I
p70S6K+
p-mTOR+
mTOR+
p-AKT
J
K
AKT+
p-PTEN
PTEN+
GAPDH
GAPDH+
Figure 1

## Slide 2
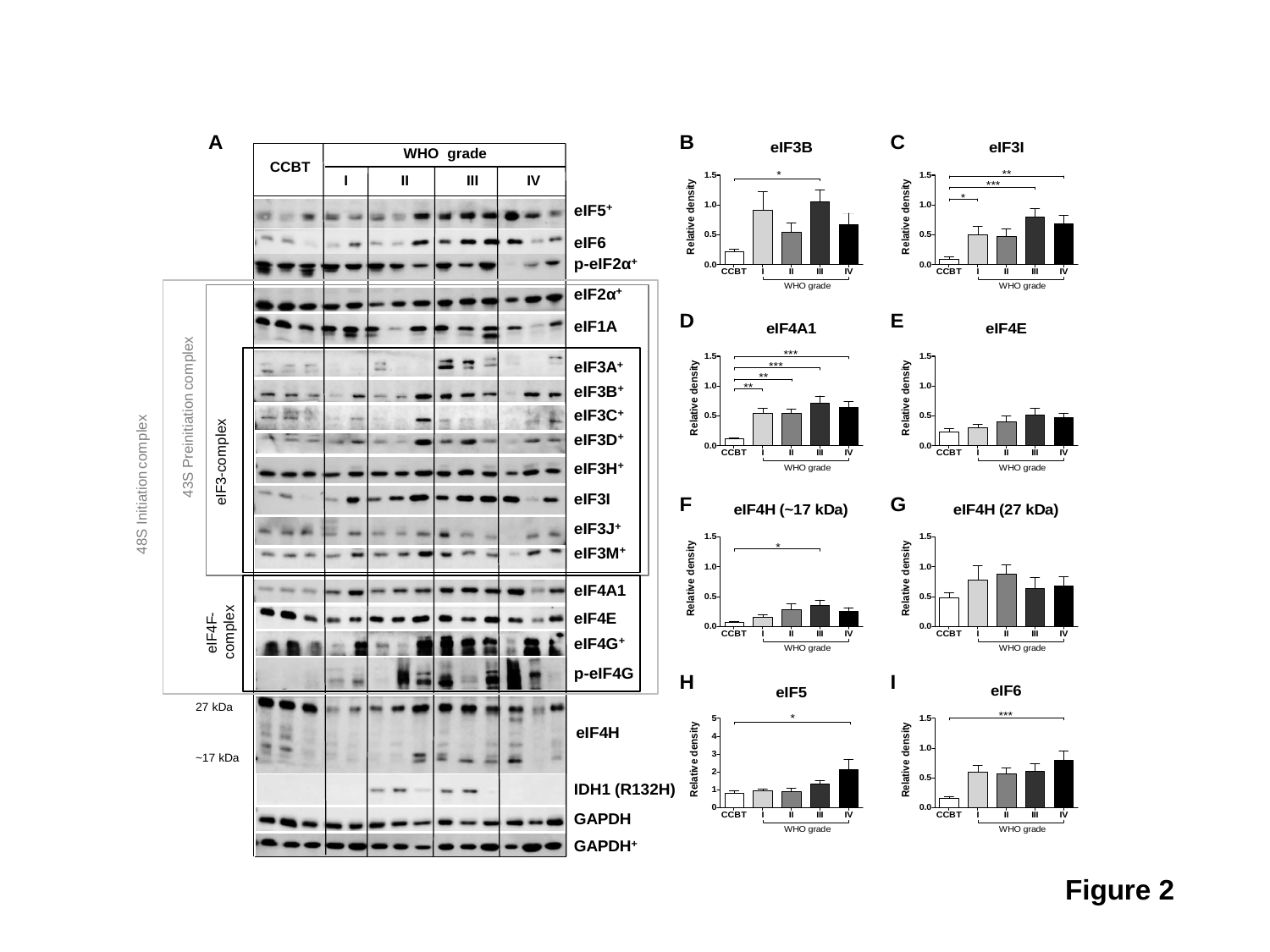

Figure 2

## Slide 3
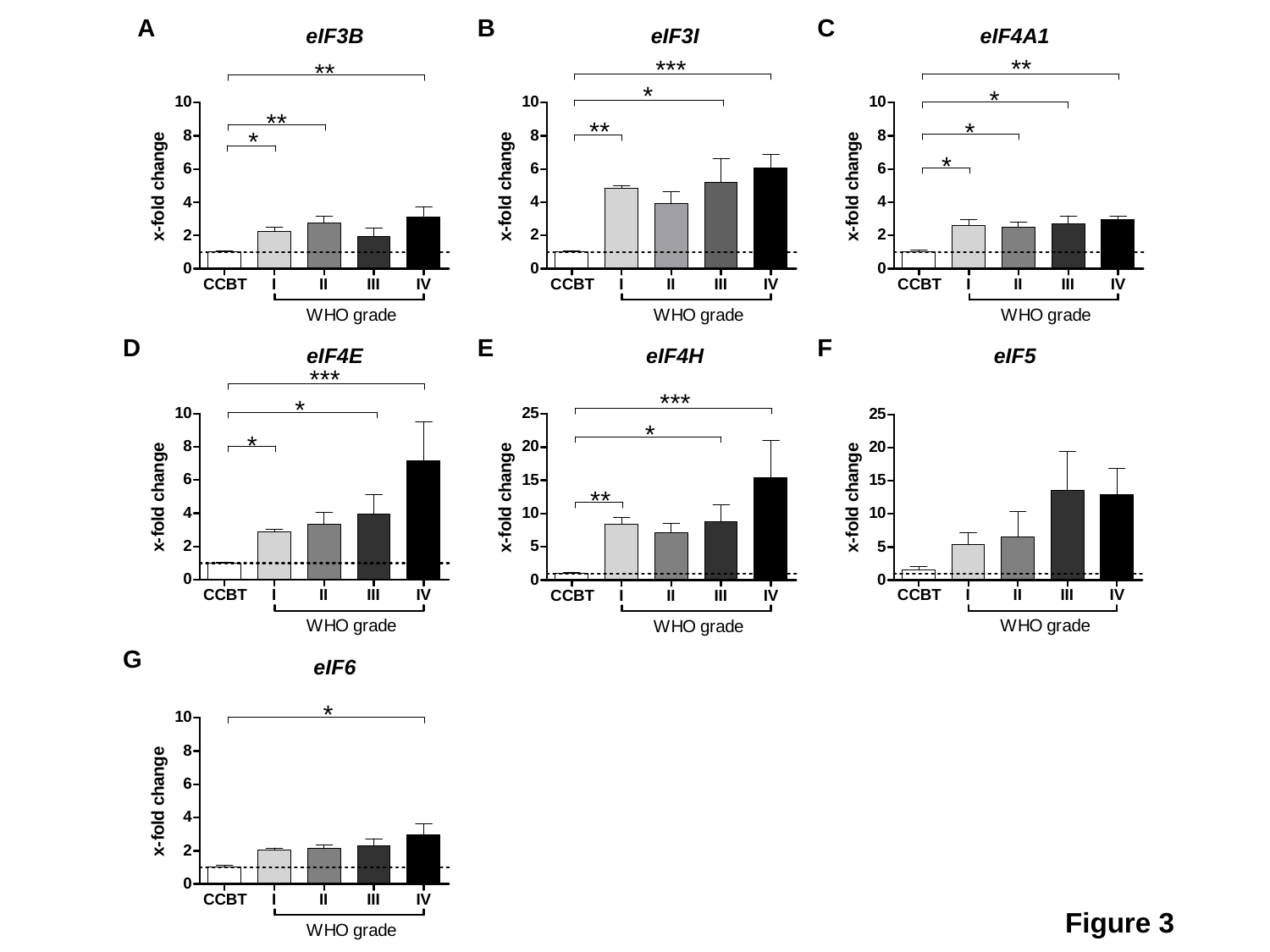

A
B
C
eIF3B
eIF3I
eIF4A1
D
E
F
eIF4E
eIF4H
eIF5
G
eIF6
Figure 3

## Slide 4
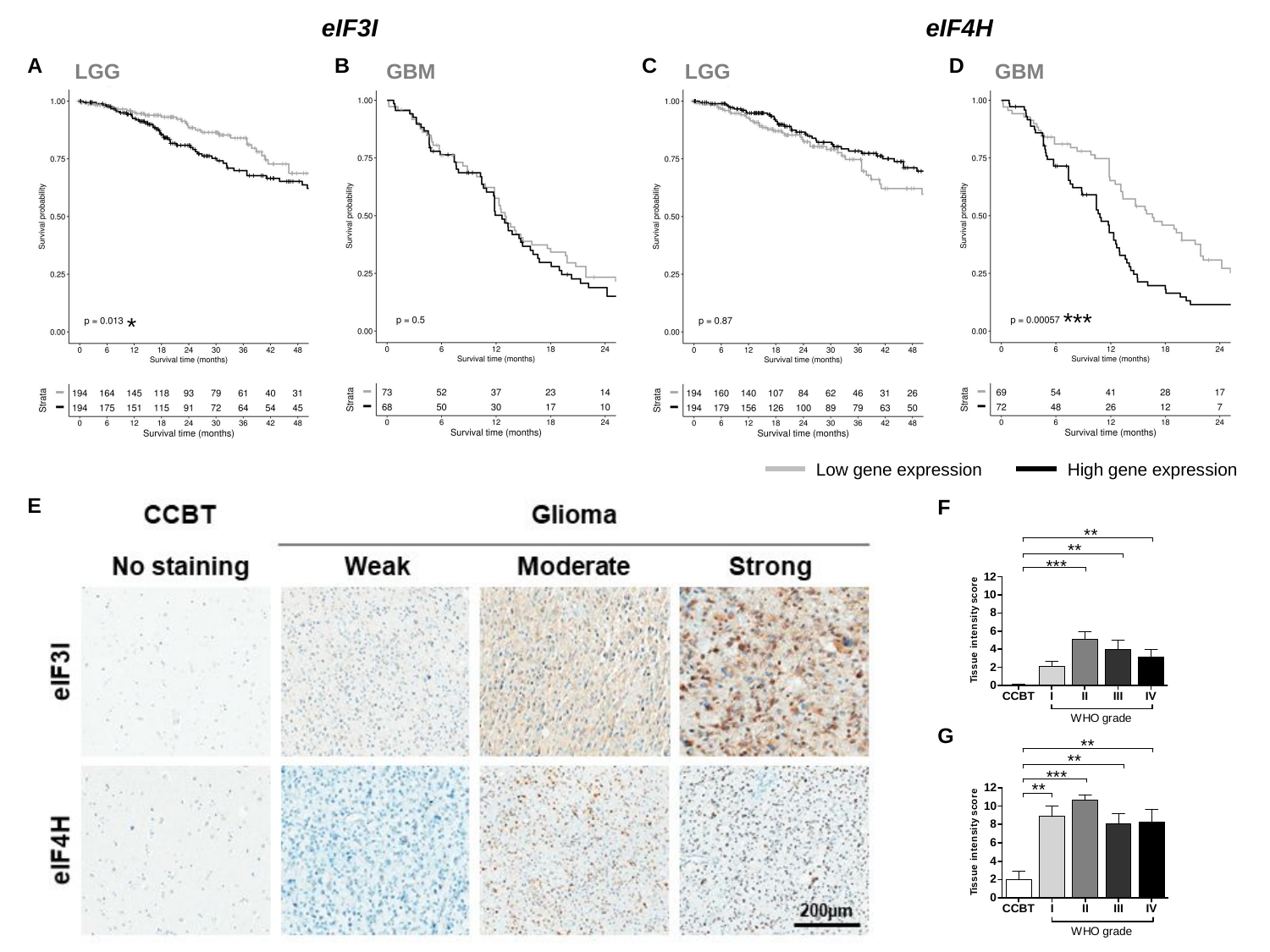

eIF3I
eIF4H
A
B
C
D
 LGG
 GBM
 LGG
 GBM
GBM
***
*
Low gene expression
High gene expression
E
F
G
Figure 4

## Slide 5
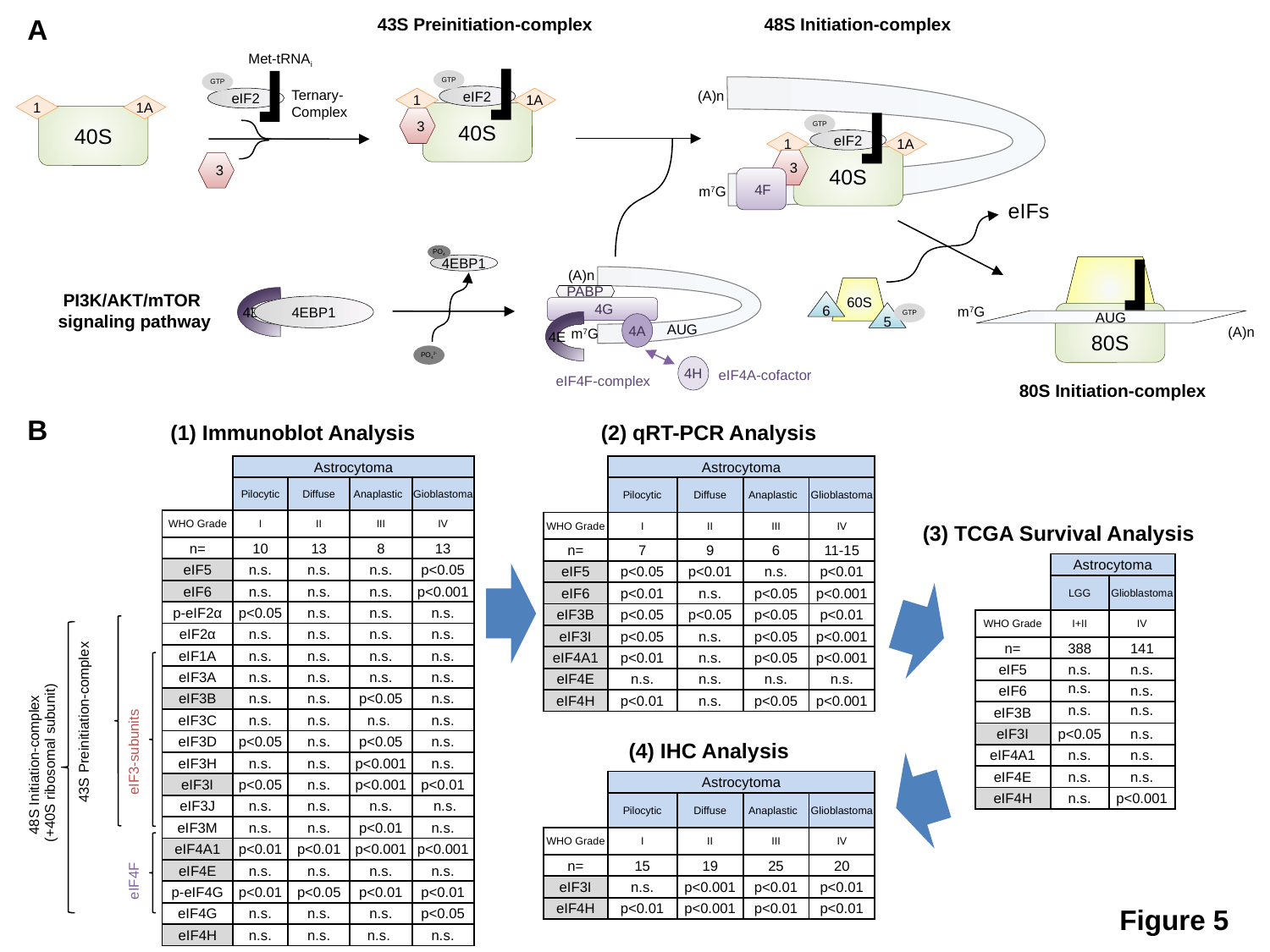

A
43S Preinitiation-complex
48S Initiation-complex
Met-tRNAi
GTP
eIF2
GTP
eIF2
1
1A
40S
Ternary-
Complex
(A)n
1
1A
40S
3
GTP
eIF2
1
1A
40S
3
3
 4F
m7G
eIFs
PO4
4EBP1
(A)n
PABP
 4G
4E
4A
AUG
m7G
eIF4F-complex
4E
60S
PI3K/AKT/mTOR
signaling pathway
6
4EBP1
m7G
AUG
5
GTP
80S
(A)n
PO42-
4H
eIF4A-cofactor
80S Initiation-complex
B
(1) Immunoblot Analysis
(2) qRT-PCR Analysis
| | | | | Astrocytoma | | | |
| --- | --- | --- | --- | --- | --- | --- | --- |
| | | | | Pilocytic | Diffuse | Anaplastic | Gioblastoma |
| | | | WHO Grade | I | II | III | IV |
| | | | n= | 10 | 13 | 8 | 13 |
| | | | eIF5 | n.s. | n.s. | n.s. | p<0.05 |
| | | | eIF6 | n.s. | n.s. | n.s. | p<0.001 |
| 48S Initiation-complex (+40S ribosomal subunit) | 43S Preinitiation-complex | | p-eIF2α | p<0.05 | n.s. | n.s. | n.s. |
| | | | eIF2α | n.s. | n.s. | n.s. | n.s. |
| | | | eIF1A | n.s. | n.s. | n.s. | n.s. |
| | | eIF3-subunits | eIF3A | n.s. | n.s. | n.s. | n.s. |
| | | | eIF3B | n.s. | n.s. | p<0.05 | n.s. |
| | | | eIF3C | n.s. | n.s. | n.s. | n.s. |
| | | | eIF3D | p<0.05 | n.s. | p<0.05 | n.s. |
| | | | eIF3H | n.s. | n.s. | p<0.001 | n.s. |
| | | | eIF3I | p<0.05 | n.s. | p<0.001 | p<0.01 |
| | | | eIF3J | n.s. | n.s. | n.s. | n.s. |
| | | | eIF3M | n.s. | n.s. | p<0.01 | n.s. |
| | | eIF4F | eIF4A1 | p<0.01 | p<0.01 | p<0.001 | p<0.001 |
| | | | eIF4E | n.s. | n.s. | n.s. | n.s. |
| | | | p-eIF4G | p<0.01 | p<0.05 | p<0.01 | p<0.01 |
| | | | eIF4G | n.s. | n.s. | n.s. | p<0.05 |
| | | | eIF4H | n.s. | n.s. | n.s. | n.s. |
| | Astrocytoma | | | |
| --- | --- | --- | --- | --- |
| | Pilocytic | Diffuse | Anaplastic | Glioblastoma |
| WHO Grade | I | II | III | IV |
| n= | 7 | 9 | 6 | 11-15 |
| eIF5 | p<0.05 | p<0.01 | n.s. | p<0.01 |
| eIF6 | p<0.01 | n.s. | p<0.05 | p<0.001 |
| eIF3B | p<0.05 | p<0.05 | p<0.05 | p<0.01 |
| eIF3I | p<0.05 | n.s. | p<0.05 | p<0.001 |
| eIF4A1 | p<0.01 | n.s. | p<0.05 | p<0.001 |
| eIF4E | n.s. | n.s. | n.s. | n.s. |
| eIF4H | p<0.01 | n.s. | p<0.05 | p<0.001 |
(3) TCGA Survival Analysis
| | Astrocytoma | |
| --- | --- | --- |
| | LGG | Glioblastoma |
| WHO Grade | I+II | IV |
| n= | 388 | 141 |
| eIF5 | n.s. | n.s. |
| eIF6 | n.s. | n.s. |
| eIF3B | n.s. | n.s. |
| eIF3I | p<0.05 | n.s. |
| eIF4A1 | n.s. | n.s. |
| eIF4E | n.s. | n.s. |
| eIF4H | n.s. | p<0.001 |
(4) IHC Analysis
| | Astrocytoma | | | |
| --- | --- | --- | --- | --- |
| | Pilocytic | Diffuse | Anaplastic | Glioblastoma |
| WHO Grade | I | II | III | IV |
| n= | 15 | 19 | 25 | 20 |
| eIF3I | n.s. | p<0.001 | p<0.01 | p<0.01 |
| eIF4H | p<0.01 | p<0.001 | p<0.01 | p<0.01 |
Figure 5

## Slide 6
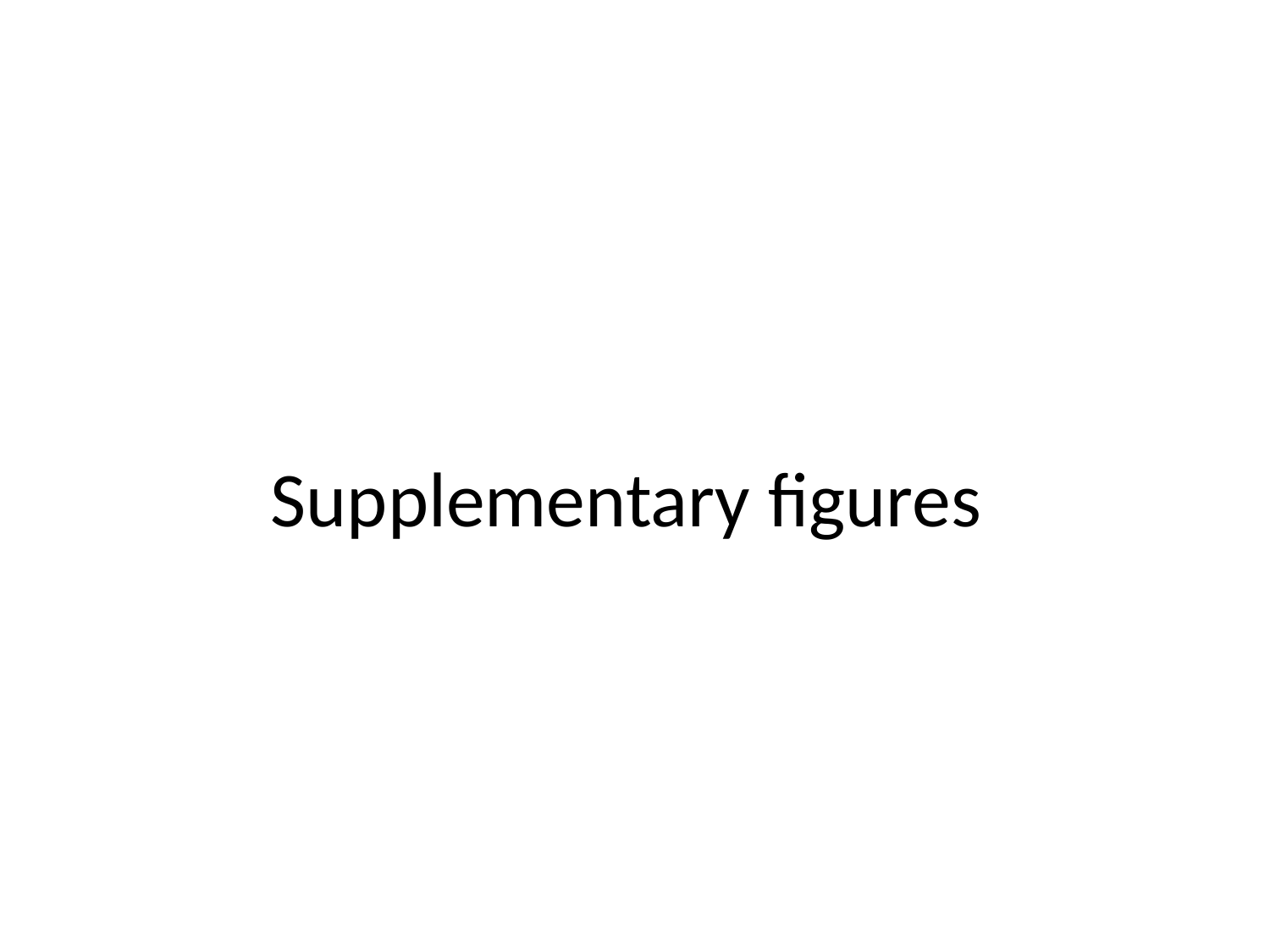

# Supplementary figures

## Slide 7
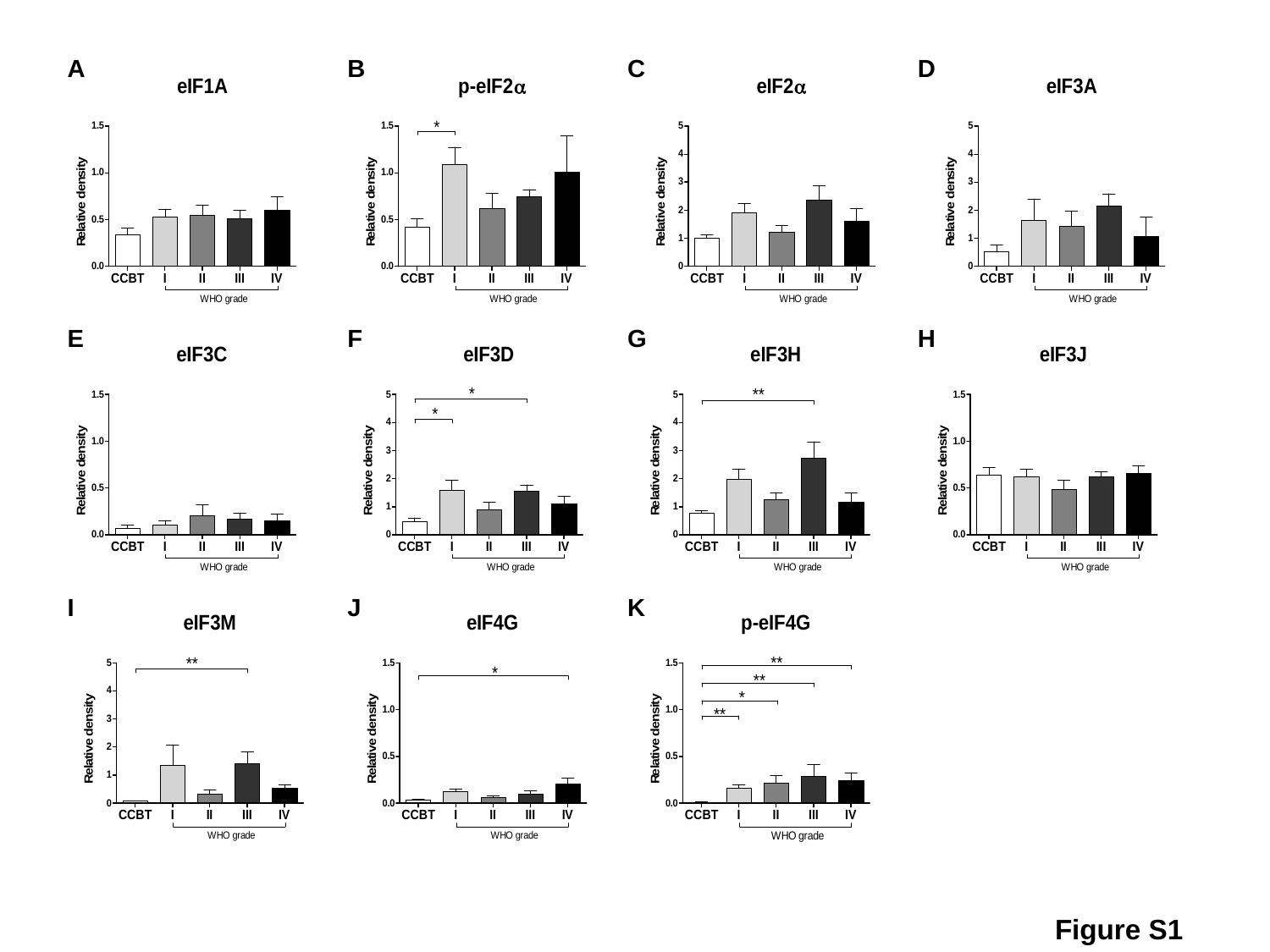

A
B
C
D
E
F
G
H
I
J
K
Figure S1

## Slide 8
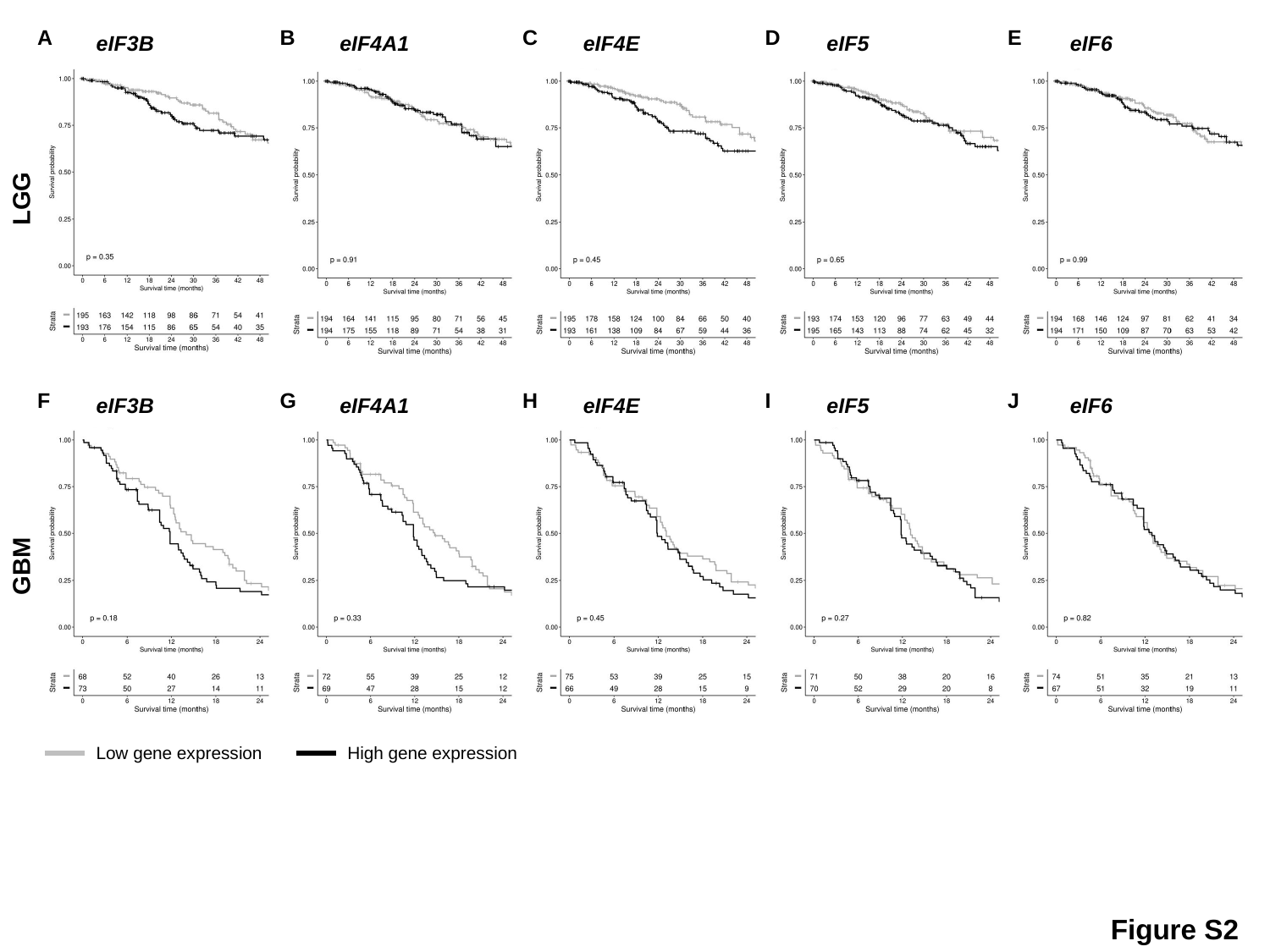

A
B
C
D
E
 eIF3B
 eIF4A1
 eIF4E
 eIF5
 eIF6
LGG
*
F
G
H
I
J
 eIF3B
 eIF4A1
 eIF4E
 eIF5
 eIF6
GBM
Low gene expression
High gene expression
Figure S2

## Slide 9
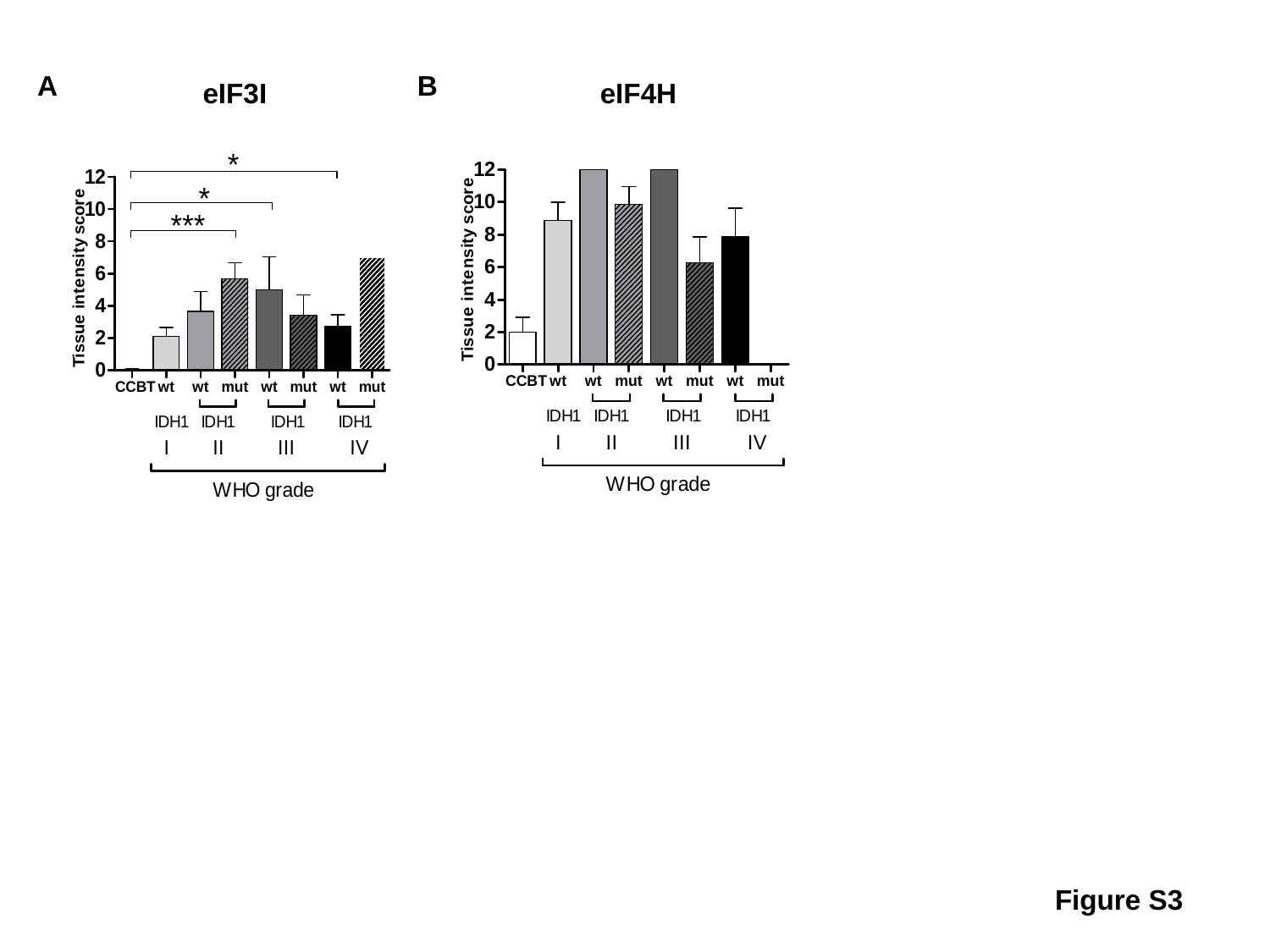

A
B
eIF3I
eIF4H
Figure S3

## Slide 10
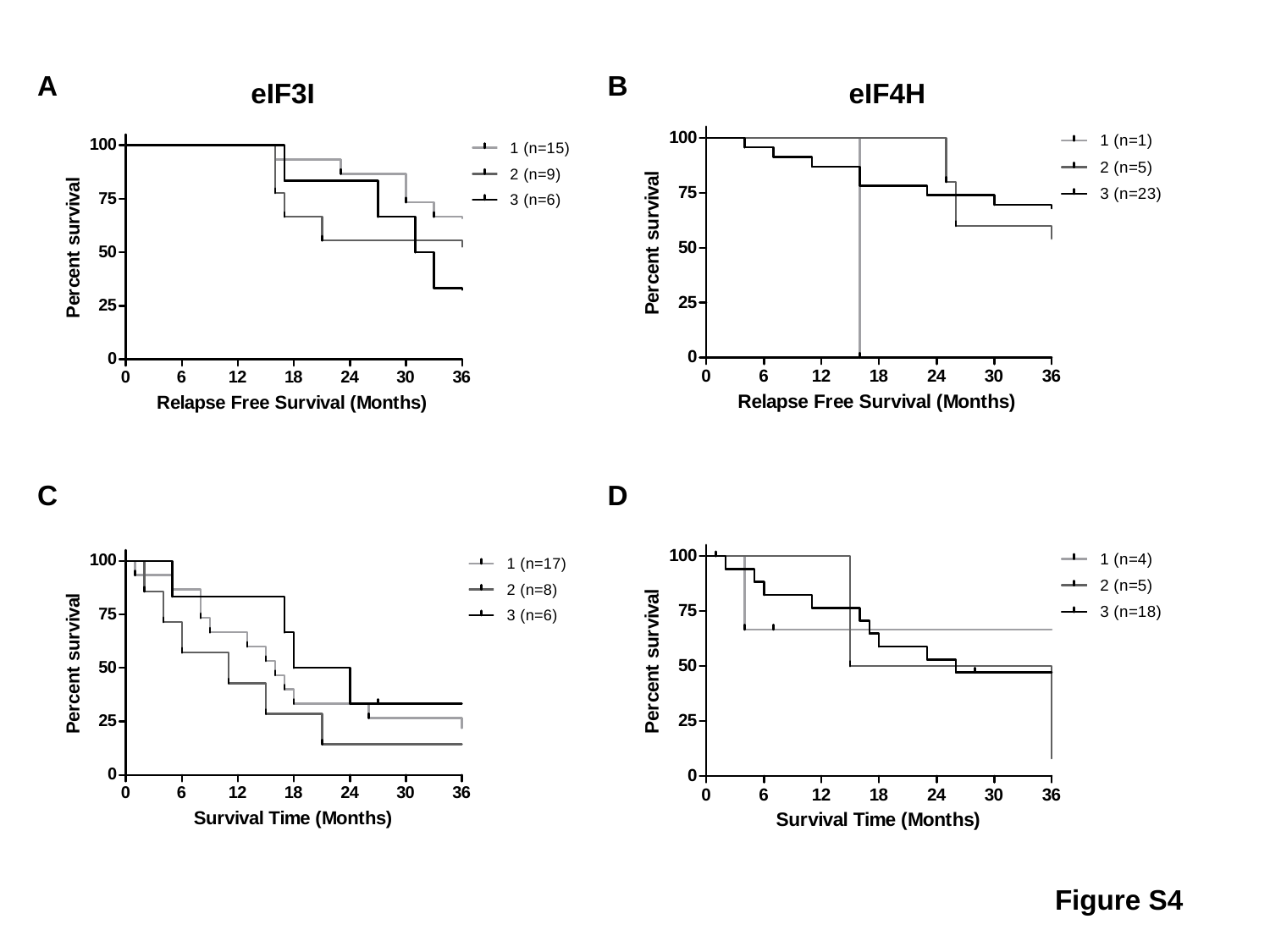

A
B
eIF3I
eIF4H
C
D
Figure S4

## Slide 11
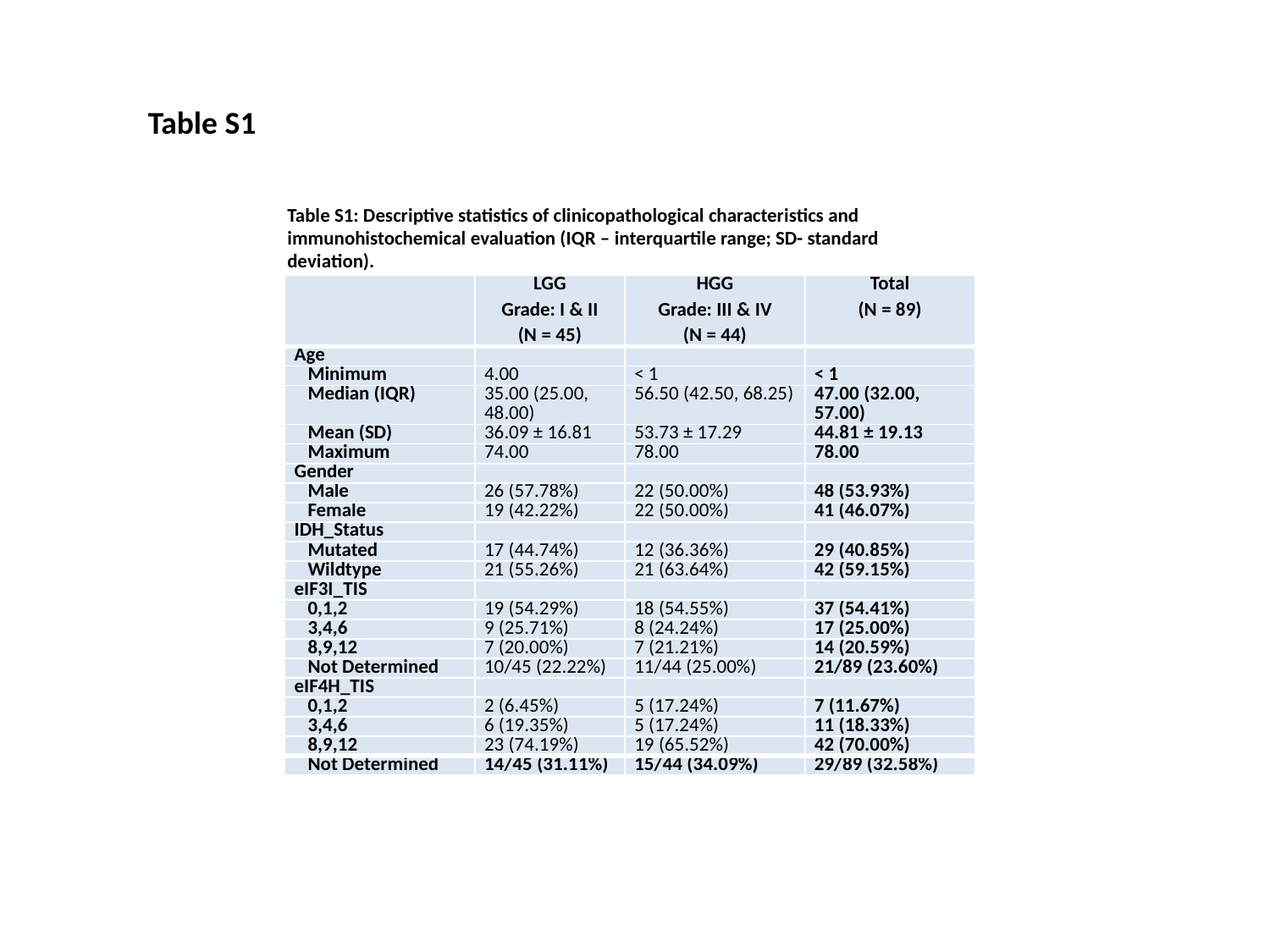

Table S1
Table S1: Descriptive statistics of clinicopathological characteristics and immunohistochemical evaluation (IQR – interquartile range; SD- standard deviation).
| | LGG Grade: I & II (N = 45) | HGG Grade: III & IV (N = 44) | Total (N = 89) |
| --- | --- | --- | --- |
| Age | | | |
| Minimum | 4.00 | < 1 | < 1 |
| Median (IQR) | 35.00 (25.00, 48.00) | 56.50 (42.50, 68.25) | 47.00 (32.00, 57.00) |
| Mean (SD) | 36.09 ± 16.81 | 53.73 ± 17.29 | 44.81 ± 19.13 |
| Maximum | 74.00 | 78.00 | 78.00 |
| Gender | | | |
| Male | 26 (57.78%) | 22 (50.00%) | 48 (53.93%) |
| Female | 19 (42.22%) | 22 (50.00%) | 41 (46.07%) |
| IDH\_Status | | | |
| Mutated | 17 (44.74%) | 12 (36.36%) | 29 (40.85%) |
| Wildtype | 21 (55.26%) | 21 (63.64%) | 42 (59.15%) |
| eIF3I\_TIS | | | |
| 0,1,2 | 19 (54.29%) | 18 (54.55%) | 37 (54.41%) |
| 3,4,6 | 9 (25.71%) | 8 (24.24%) | 17 (25.00%) |
| 8,9,12 | 7 (20.00%) | 7 (21.21%) | 14 (20.59%) |
| Not Determined | 10/45 (22.22%) | 11/44 (25.00%) | 21/89 (23.60%) |
| eIF4H\_TIS | | | |
| 0,1,2 | 2 (6.45%) | 5 (17.24%) | 7 (11.67%) |
| 3,4,6 | 6 (19.35%) | 5 (17.24%) | 11 (18.33%) |
| 8,9,12 | 23 (74.19%) | 19 (65.52%) | 42 (70.00%) |
| Not Determined | 14/45 (31.11%) | 15/44 (34.09%) | 29/89 (32.58%) |

## Slide 12
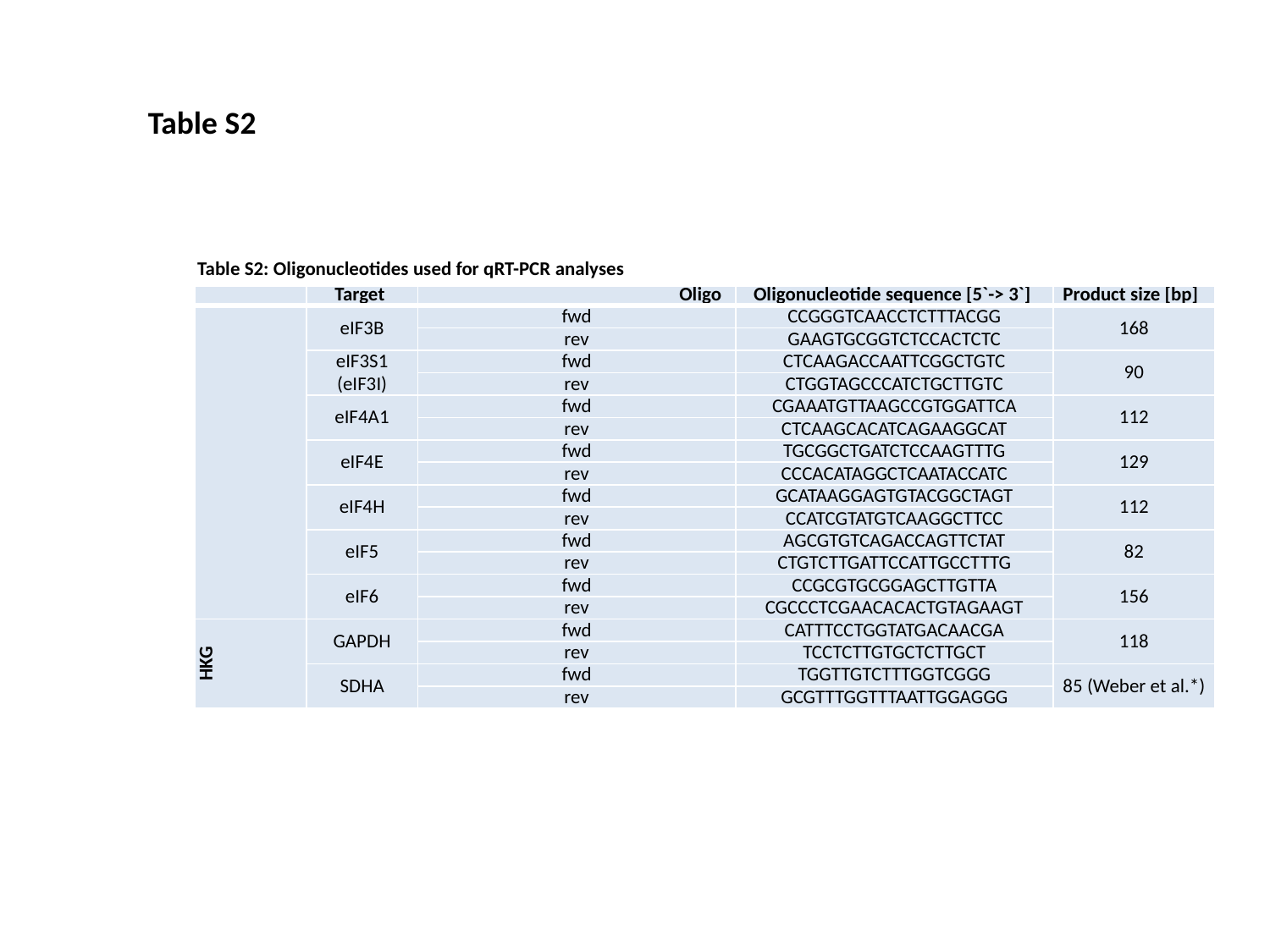

Table S2
Table S2: Oligonucleotides used for qRT-PCR analyses
| | Target | Oligo | Oligonucleotide sequence [5`-> 3`] | Product size [bp] |
| --- | --- | --- | --- | --- |
| | eIF3B | fwd | CCGGGTCAACCTCTTTACGG | 168 |
| | | rev | GAAGTGCGGTCTCCACTCTC | |
| | eIF3S1 (eIF3I) | fwd | CTCAAGACCAATTCGGCTGTC | 90 |
| | | rev | CTGGTAGCCCATCTGCTTGTC | |
| | eIF4A1 | fwd | CGAAATGTTAAGCCGTGGATTCA | 112 |
| | | rev | CTCAAGCACATCAGAAGGCAT | |
| | eIF4E | fwd | TGCGGCTGATCTCCAAGTTTG | 129 |
| | | rev | CCCACATAGGCTCAATACCATC | |
| | eIF4H | fwd | GCATAAGGAGTGTACGGCTAGT | 112 |
| | | rev | CCATCGTATGTCAAGGCTTCC | |
| | eIF5 | fwd | AGCGTGTCAGACCAGTTCTAT | 82 |
| | | rev | CTGTCTTGATTCCATTGCCTTTG | |
| | eIF6 | fwd | CCGCGTGCGGAGCTTGTTA | 156 |
| | | rev | CGCCCTCGAACACACTGTAGAAGT | |
| HKG | GAPDH | fwd | CATTTCCTGGTATGACAACGA | 118 |
| | | rev | TCCTCTTGTGCTCTTGCT | |
| | SDHA | fwd | TGGTTGTCTTTGGTCGGG | 85 (Weber et al.\*) |
| | | rev | GCGTTTGGTTTAATTGGAGGG | |

## Slide 13
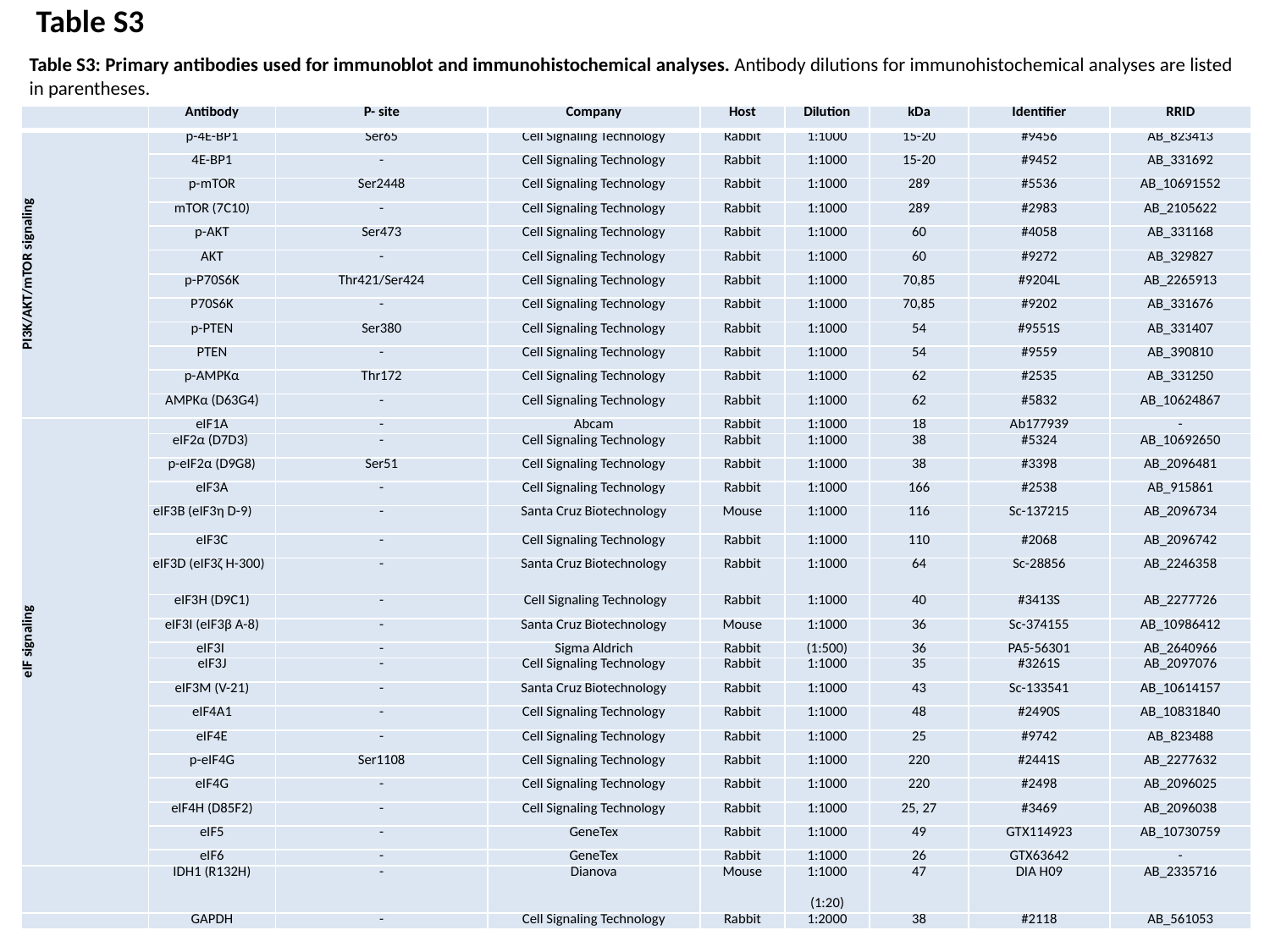

Table S3
Table S3: Primary antibodies used for immunoblot and immunohistochemical analyses. Antibody dilutions for immunohistochemical analyses are listed in parentheses.
| | Antibody | P- site | Company | Host | Dilution | kDa | Identifier | RRID |
| --- | --- | --- | --- | --- | --- | --- | --- | --- |
| PI3K/AKT/mTOR signaling | p-4E-BP1 | Ser65 | Cell Signaling Technology | Rabbit | 1:1000 | 15-20 | #9456 | AB\_823413 |
| | 4E-BP1 | - | Cell Signaling Technology | Rabbit | 1:1000 | 15-20 | #9452 | AB\_331692 |
| | p-mTOR | Ser2448 | Cell Signaling Technology | Rabbit | 1:1000 | 289 | #5536 | AB\_10691552 |
| | mTOR (7C10) | - | Cell Signaling Technology | Rabbit | 1:1000 | 289 | #2983 | AB\_2105622 |
| | p-AKT | Ser473 | Cell Signaling Technology | Rabbit | 1:1000 | 60 | #4058 | AB\_331168 |
| | AKT | - | Cell Signaling Technology | Rabbit | 1:1000 | 60 | #9272 | AB\_329827 |
| | p-P70S6K | Thr421/Ser424 | Cell Signaling Technology | Rabbit | 1:1000 | 70,85 | #9204L | AB\_2265913 |
| | P70S6K | - | Cell Signaling Technology | Rabbit | 1:1000 | 70,85 | #9202 | AB\_331676 |
| | p-PTEN | Ser380 | Cell Signaling Technology | Rabbit | 1:1000 | 54 | #9551S | AB\_331407 |
| | PTEN | - | Cell Signaling Technology | Rabbit | 1:1000 | 54 | #9559 | AB\_390810 |
| | p-AMPKα | Thr172 | Cell Signaling Technology | Rabbit | 1:1000 | 62 | #2535 | AB\_331250 |
| | AMPKα (D63G4) | - | Cell Signaling Technology | Rabbit | 1:1000 | 62 | #5832 | AB\_10624867 |
| eIF signaling | eIF1A | - | Abcam | Rabbit | 1:1000 | 18 | Ab177939 | - |
| | eIF2α (D7D3) | - | Cell Signaling Technology | Rabbit | 1:1000 | 38 | #5324 | AB\_10692650 |
| | p-eIF2α (D9G8) | Ser51 | Cell Signaling Technology | Rabbit | 1:1000 | 38 | #3398 | AB\_2096481 |
| | eIF3A | - | Cell Signaling Technology | Rabbit | 1:1000 | 166 | #2538 | AB\_915861 |
| | eIF3B (eIF3η D-9) | - | Santa Cruz Biotechnology | Mouse | 1:1000 | 116 | Sc-137215 | AB\_2096734 |
| | eIF3C | - | Cell Signaling Technology | Rabbit | 1:1000 | 110 | #2068 | AB\_2096742 |
| | eIF3D (eIF3ζ H-300) | - | Santa Cruz Biotechnology | Rabbit | 1:1000 | 64 | Sc-28856 | AB\_2246358 |
| | eIF3H (D9C1) | - | Cell Signaling Technology | Rabbit | 1:1000 | 40 | #3413S | AB\_2277726 |
| | eIF3I (eIF3β A-8) | - | Santa Cruz Biotechnology | Mouse | 1:1000 | 36 | Sc-374155 | AB\_10986412 |
| | eIF3I | - | Sigma Aldrich | Rabbit | (1:500) | 36 | PA5-56301 | AB\_2640966 |
| | eIF3J | - | Cell Signaling Technology | Rabbit | 1:1000 | 35 | #3261S | AB\_2097076 |
| | eIF3M (V-21) | - | Santa Cruz Biotechnology | Rabbit | 1:1000 | 43 | Sc-133541 | AB\_10614157 |
| | eIF4A1 | - | Cell Signaling Technology | Rabbit | 1:1000 | 48 | #2490S | AB\_10831840 |
| | eIF4E | - | Cell Signaling Technology | Rabbit | 1:1000 | 25 | #9742 | AB\_823488 |
| | p-eIF4G | Ser1108 | Cell Signaling Technology | Rabbit | 1:1000 | 220 | #2441S | AB\_2277632 |
| | eIF4G | - | Cell Signaling Technology | Rabbit | 1:1000 | 220 | #2498 | AB\_2096025 |
| | eIF4H (D85F2) | - | Cell Signaling Technology | Rabbit | 1:1000 | 25, 27 | #3469 | AB\_2096038 |
| | eIF5 | - | GeneTex | Rabbit | 1:1000 | 49 | GTX114923 | AB\_10730759 |
| | eIF6 | - | GeneTex | Rabbit | 1:1000 | 26 | GTX63642 | - |
| | IDH1 (R132H) | - | Dianova | Mouse | 1:1000 (1:20) | 47 | DIA H09 | AB\_2335716 |
| | GAPDH | - | Cell Signaling Technology | Rabbit | 1:2000 | 38 | #2118 | AB\_561053 |

## Slide 14
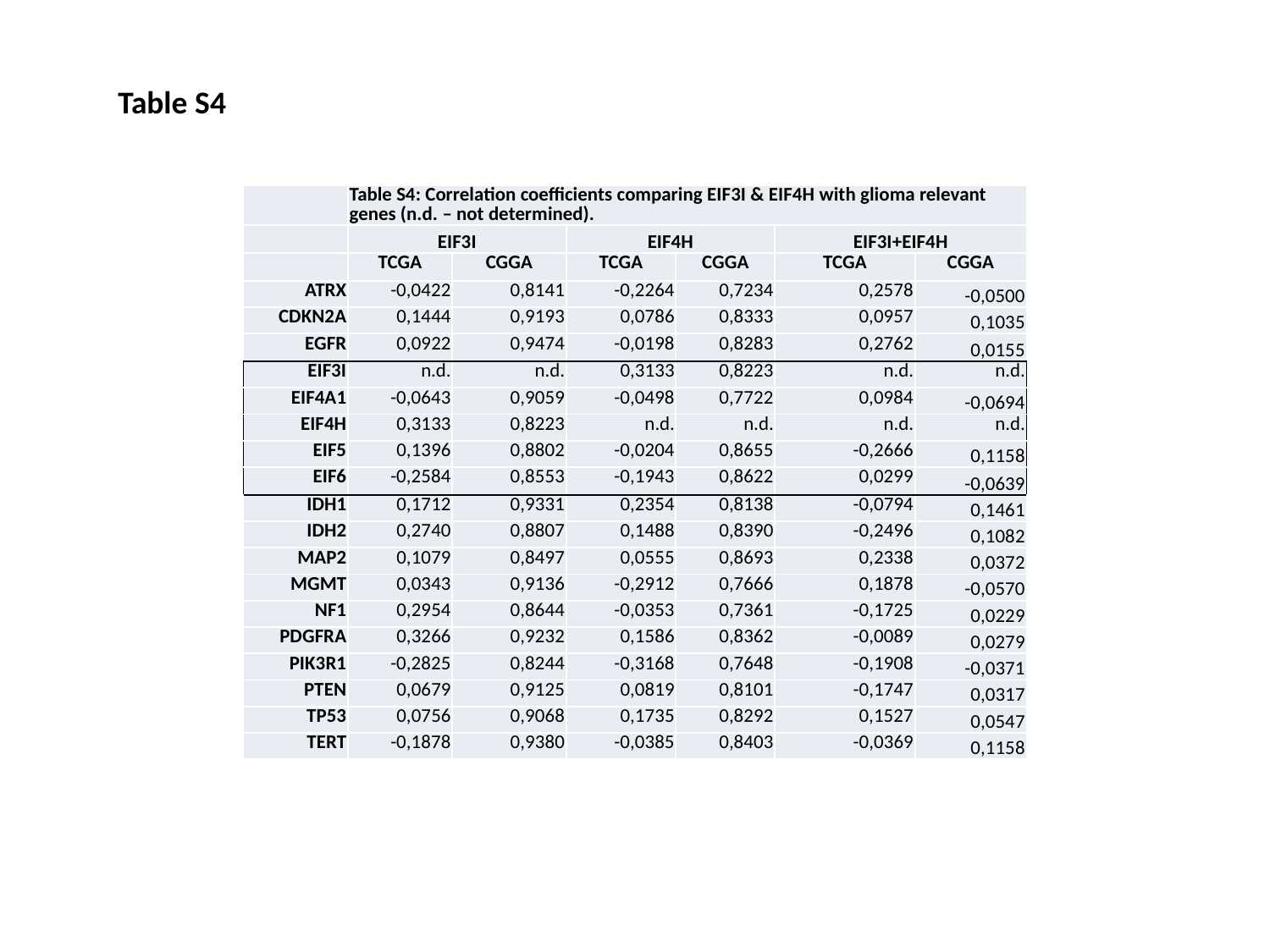

Table S4
| | Table S4: Correlation coefficients comparing EIF3I & EIF4H with glioma relevant genes (n.d. – not determined). | | | | | |
| --- | --- | --- | --- | --- | --- | --- |
| | EIF3I | | EIF4H | | EIF3I+EIF4H | |
| | TCGA | CGGA | TCGA | CGGA | TCGA | CGGA |
| ATRX | -0,0422 | 0,8141 | -0,2264 | 0,7234 | 0,2578 | -0,0500 |
| CDKN2A | 0,1444 | 0,9193 | 0,0786 | 0,8333 | 0,0957 | 0,1035 |
| EGFR | 0,0922 | 0,9474 | -0,0198 | 0,8283 | 0,2762 | 0,0155 |
| EIF3I | n.d. | n.d. | 0,3133 | 0,8223 | n.d. | n.d. |
| EIF4A1 | -0,0643 | 0,9059 | -0,0498 | 0,7722 | 0,0984 | -0,0694 |
| EIF4H | 0,3133 | 0,8223 | n.d. | n.d. | n.d. | n.d. |
| EIF5 | 0,1396 | 0,8802 | -0,0204 | 0,8655 | -0,2666 | 0,1158 |
| EIF6 | -0,2584 | 0,8553 | -0,1943 | 0,8622 | 0,0299 | -0,0639 |
| IDH1 | 0,1712 | 0,9331 | 0,2354 | 0,8138 | -0,0794 | 0,1461 |
| IDH2 | 0,2740 | 0,8807 | 0,1488 | 0,8390 | -0,2496 | 0,1082 |
| MAP2 | 0,1079 | 0,8497 | 0,0555 | 0,8693 | 0,2338 | 0,0372 |
| MGMT | 0,0343 | 0,9136 | -0,2912 | 0,7666 | 0,1878 | -0,0570 |
| NF1 | 0,2954 | 0,8644 | -0,0353 | 0,7361 | -0,1725 | 0,0229 |
| PDGFRA | 0,3266 | 0,9232 | 0,1586 | 0,8362 | -0,0089 | 0,0279 |
| PIK3R1 | -0,2825 | 0,8244 | -0,3168 | 0,7648 | -0,1908 | -0,0371 |
| PTEN | 0,0679 | 0,9125 | 0,0819 | 0,8101 | -0,1747 | 0,0317 |
| TP53 | 0,0756 | 0,9068 | 0,1735 | 0,8292 | 0,1527 | 0,0547 |
| TERT | -0,1878 | 0,9380 | -0,0385 | 0,8403 | -0,0369 | 0,1158 |

## Slide 15
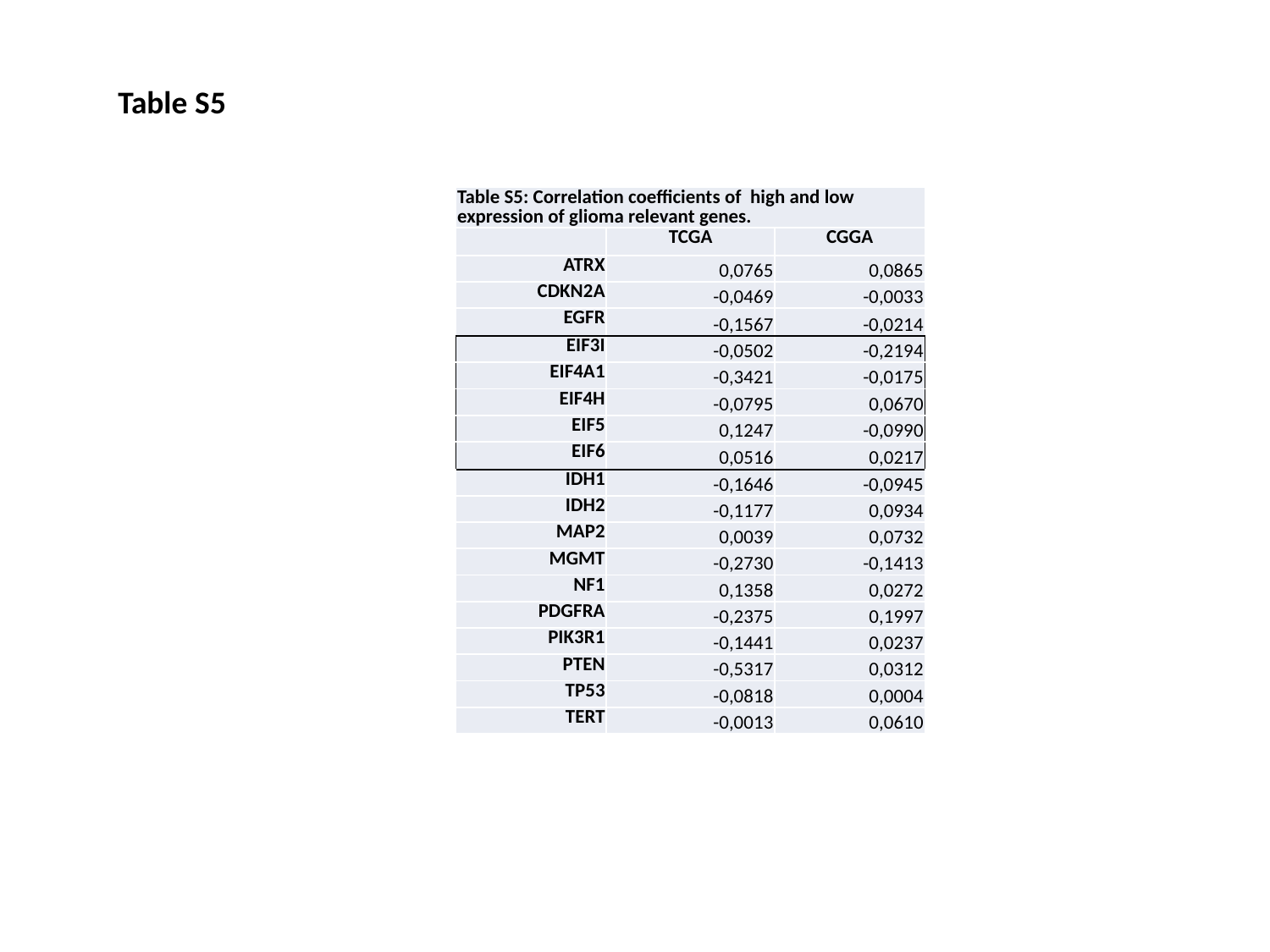

Table S5
| Table S5: Correlation coefficients of high and low expression of glioma relevant genes. | | |
| --- | --- | --- |
| | TCGA | CGGA |
| ATRX | 0,0765 | 0,0865 |
| CDKN2A | -0,0469 | -0,0033 |
| EGFR | -0,1567 | -0,0214 |
| EIF3I | -0,0502 | -0,2194 |
| EIF4A1 | -0,3421 | -0,0175 |
| EIF4H | -0,0795 | 0,0670 |
| EIF5 | 0,1247 | -0,0990 |
| EIF6 | 0,0516 | 0,0217 |
| IDH1 | -0,1646 | -0,0945 |
| IDH2 | -0,1177 | 0,0934 |
| MAP2 | 0,0039 | 0,0732 |
| MGMT | -0,2730 | -0,1413 |
| NF1 | 0,1358 | 0,0272 |
| PDGFRA | -0,2375 | 0,1997 |
| PIK3R1 | -0,1441 | 0,0237 |
| PTEN | -0,5317 | 0,0312 |
| TP53 | -0,0818 | 0,0004 |
| TERT | -0,0013 | 0,0610 |

## Slide 16
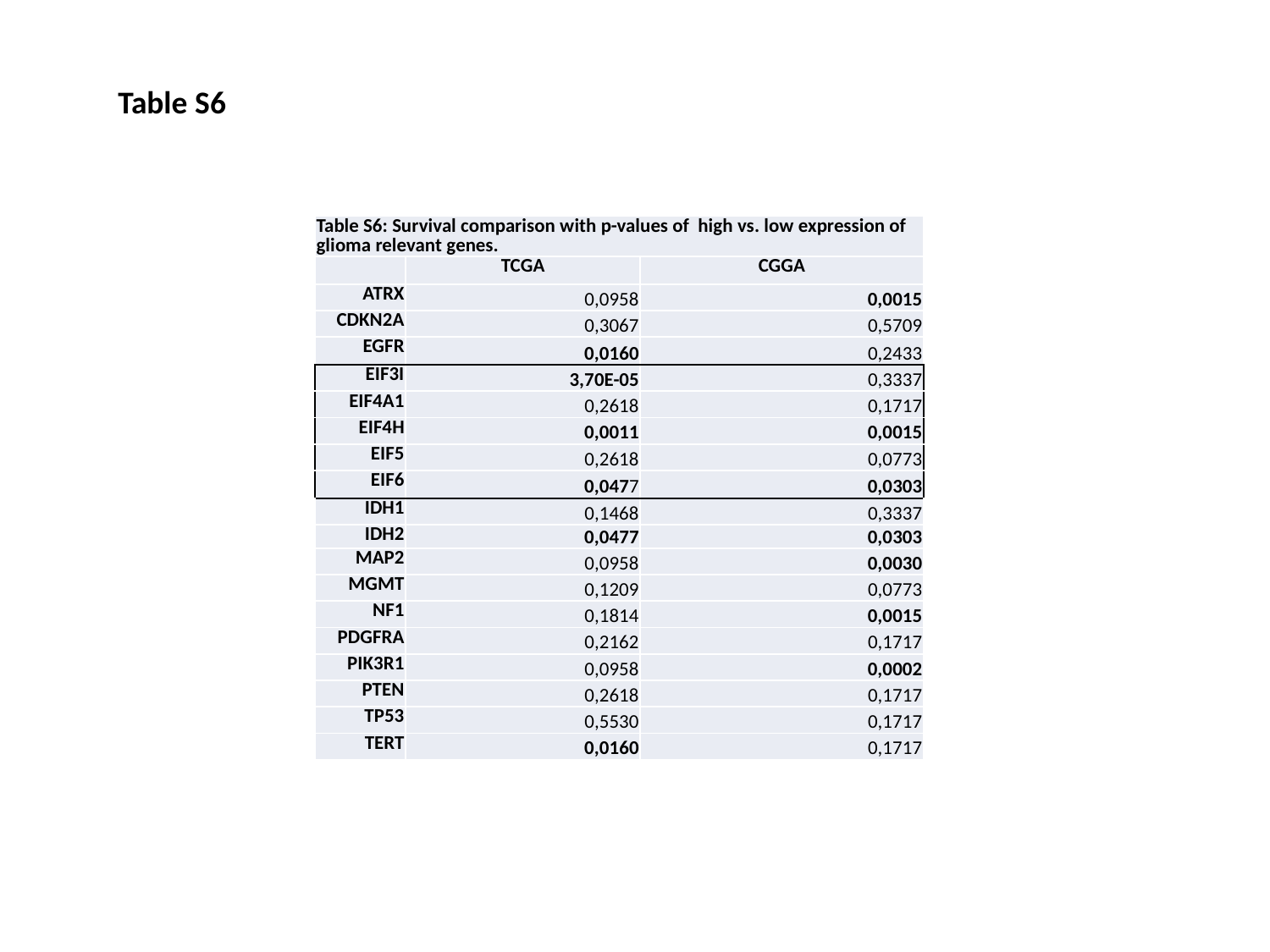

Table S6
| Table S6: Survival comparison with p-values of high vs. low expression of glioma relevant genes. | | |
| --- | --- | --- |
| | TCGA | CGGA |
| ATRX | 0,0958 | 0,0015 |
| CDKN2A | 0,3067 | 0,5709 |
| EGFR | 0,0160 | 0,2433 |
| EIF3I | 3,70E-05 | 0,3337 |
| EIF4A1 | 0,2618 | 0,1717 |
| EIF4H | 0,0011 | 0,0015 |
| EIF5 | 0,2618 | 0,0773 |
| EIF6 | 0,0477 | 0,0303 |
| IDH1 | 0,1468 | 0,3337 |
| IDH2 | 0,0477 | 0,0303 |
| MAP2 | 0,0958 | 0,0030 |
| MGMT | 0,1209 | 0,0773 |
| NF1 | 0,1814 | 0,0015 |
| PDGFRA | 0,2162 | 0,1717 |
| PIK3R1 | 0,0958 | 0,0002 |
| PTEN | 0,2618 | 0,1717 |
| TP53 | 0,5530 | 0,1717 |
| TERT | 0,0160 | 0,1717 |
